# Computational identification of host genomic biomarkers highlighting their functions, pathways and regulators that influence SARS-CoV-2 infections and drug repurposing

**DOI:** 10.1038/s41598-022-08073-8

**Published:** 2022-03-11

**Authors:** Md. Parvez Mosharaf, Md. Selim Reza, Md. Kaderi Kibria, Fee Faysal Ahmed, Md. Hadiul Kabir, Sohel Hasan, Md. Nurul Haque Mollah

**Affiliations:** 1 Bioinformatics Laboratory, Department of Statistics, Rajshahi University, Rajshahi, 6205 Bangladesh; 2 Department of Mathematics, Jashore University of Science and Technology, Jashore, Bangladesh; 3 Department of Biochemistry and Molecular Biology, Rajshahi University, Rajshahi, 6205 Bangladesh; 4 School of Commerce, Faculty of Business, Education, Law and Arts, University of Southern Queensland, Toowoomba, QLD 4350 Australia

**Keywords:** Computational biology and bioinformatics, Drug discovery, Systems biology

## Abstract

The pandemic threat of COVID-19 has severely destroyed human life as well as the economy around the world. Although, the vaccination has reduced the outspread, but people are still suffering due to the unstable RNA sequence patterns of SARS-CoV-2 which demands supplementary drugs. To explore novel drug target proteins, in this study, a transcriptomics RNA-Seq data generated from SARS-CoV-2 infection and control samples were analyzed. We identified 109 differentially expressed genes (DEGs) that were utilized to identify 10 hub-genes/proteins (TLR2, USP53, GUCY1A2, SNRPD2, NEDD9, IGF2, CXCL2, KLF6, PAG1 and ZFP36) by the protein–protein interaction (PPI) network analysis. The GO functional and KEGG pathway enrichment analyses of hub-DEGs revealed some important functions and signaling pathways that are significantly associated with SARS-CoV-2 infections. The interaction network analysis identified 5 TFs proteins and 6 miRNAs as the key regulators of hub-DEGs. Considering 10 hub-proteins and 5 key TFs-proteins as drug target receptors, we performed their docking analysis with the SARS-CoV-2 3CL protease-guided top listed 90 FDA approved drugs. We found Torin-2, Rapamycin, Radotinib, Ivermectin, Thiostrepton, Tacrolimus and Daclatasvir as the top ranked seven candidate drugs. We investigated their resistance performance against the already published COVID-19 causing top-ranked 11 independent and 8 protonated receptor proteins by molecular docking analysis and found their strong binding affinities, which indicates that the proposed drugs are effective against the state-of-the-arts alternatives independent receptor proteins also. Finally, we investigated the stability of top three drugs (Torin-2, Rapamycin and Radotinib) by using 100 ns MD-based MM-PBSA simulations with the two top-ranked proposed receptors (TLR2, USP53) and independent receptors (IRF7, STAT1), and observed their stable performance. Therefore, the proposed drugs might play a vital role for the treatment against different variants of SARS-CoV-2 infections.

## Introduction

The severe acute respiratory syndrome coronavirus (SARS-CoV) is an alarming global health concern starting from the early twenty-first century. This virus is named Coronaviruses (CoVs) because of its characteristic halo structure under an electron microscope (corona, crown-like). Latin word “corona” means crown or “halo” and coronavirus particles display a crown-like fringe typically referred to as “spikes” under electron microscopy^1^. The CoVs are non-segmented single-stranded RNA viruses covered with envelope which can cause illness ranging in severity from the common cold to severe and fatal illness or even death. On the basis of serotype and genome, the coronavirus subfamily is divided into four genera: α, β, γ and δ, which have long been recognized as important veterinary pathogens that cause severe to lethal respiratory and enteric diseases in birds as well as mammals. Consequently, the SARS-CoV is a severe respiratory tract disease which was originally distinguished in Guangdong Province, China in 2002 and afterward it spread to 29 countries and was first authoritatively perceived in March 2003^2^. In excess of 8,000 likely cases and 774 deaths were accounted for worldwide between March 2003 and July 2003 because of the outbreak of this coronavirus (CoV)^3^. During the outbreak, the normal death rate was around 9.6 percent^4,5^. Koch's proposed that SARS-CoV was identified with pathogenesis and it is a huge danger to human health^6^. Moreover, Acute Respiratory Distress Syndrome (ARDS) was found in 16% of the all-out SARS-CoV patients and the death rate became half in case of these kinds of SARS-CoV patients^7,8^. The year 2020 was started with the alarming of SARS-CoV-2 infections that has outbreak the novel coronavirus disease 2019 (COVID-19) and become a global pandemic. It has genetic similarity with SARS-CoV^9^. The COVID-19 causing a massive loss of lives was formally announced a pandemic by the WHO on 11 March, 2020^10^. The COVID-19 affected persons suffer from fever, shortness of breath and cough within an incubation period of 2–14 days with both symptomatic and asymptomatic symptoms^9^. At present, the COVID-19 pandemic is a deadly and dangerous health concern around the globe. Coronaviruses are single-stranded, positive-sense RNA virus which has huge genomes of viral RNA^11^. Current examinations revealed that SARS-CoV-2 has a genomic structure near that of other beta-coronaviruses^12^. The novel Corona virus-2 makes a contrast in homogeneity with MERS-CoV and SARS-CoV while it has been sorted with the beta-coronaviruses. However, the SARS-CoV-2 genes seem to be as 89.10% nucleotide likeliness as well as < 80% nucleotide uniformity compared to the SARS-CoV genes^13,14^. SARS-CoV-2 has been identified as the seventh known human coronavirus (HCoV) from a similar family after 229E, NL63, OC43, HKU1, MERS-CoV and SARS-CoV^15^.

Nevertheless, the destructive stream/wave of SARS-CoV-2 to human life that causes millions of lives loss globally along with other paralyzing effects on economy as well as human civilization is unparallel and is close to every door. Almost all the countries are affected by this devastating wave but the United States, Brazil, Italy, Russia, Spain, UK and India are among the top six countries where SARS-CoV-2 has spread the most according to the report of Worldometer (https://www.worldometers.info/coronavirus/). As of 16 June 2021, around 4 million peoples died out of 175 million SARS-CoV-2 infections and gradually infected peoples are increasing worldwide. Scientists and pharmaceutical companies around the globe are trying to find out the way of escape from this devastating pandemic situation by means of discovering drugs and effective vaccines against COVID-19 virus. According to the published report in GISAID (Global Initiative on Sharing All Influenza Data) database on 20 November 2020, the SARS-CoV-2 strains were clustered into 8 clades (S, L, V, G, GR, GH, GV and others) based on their RNA sequence patterns and gradually clades are increasing. The unstable patterns of RNA sequences make this virus infections untouched and uncontrolled because once vaccine is going to be prepared based on a sequence of SARS-CoV-2, but afterwards this virus is automatically changing its genomic structure by mutations which through all the efforts in vein. Indeed, the mechanism is mysterious and horrible to control this virus.

**Figure 1:**
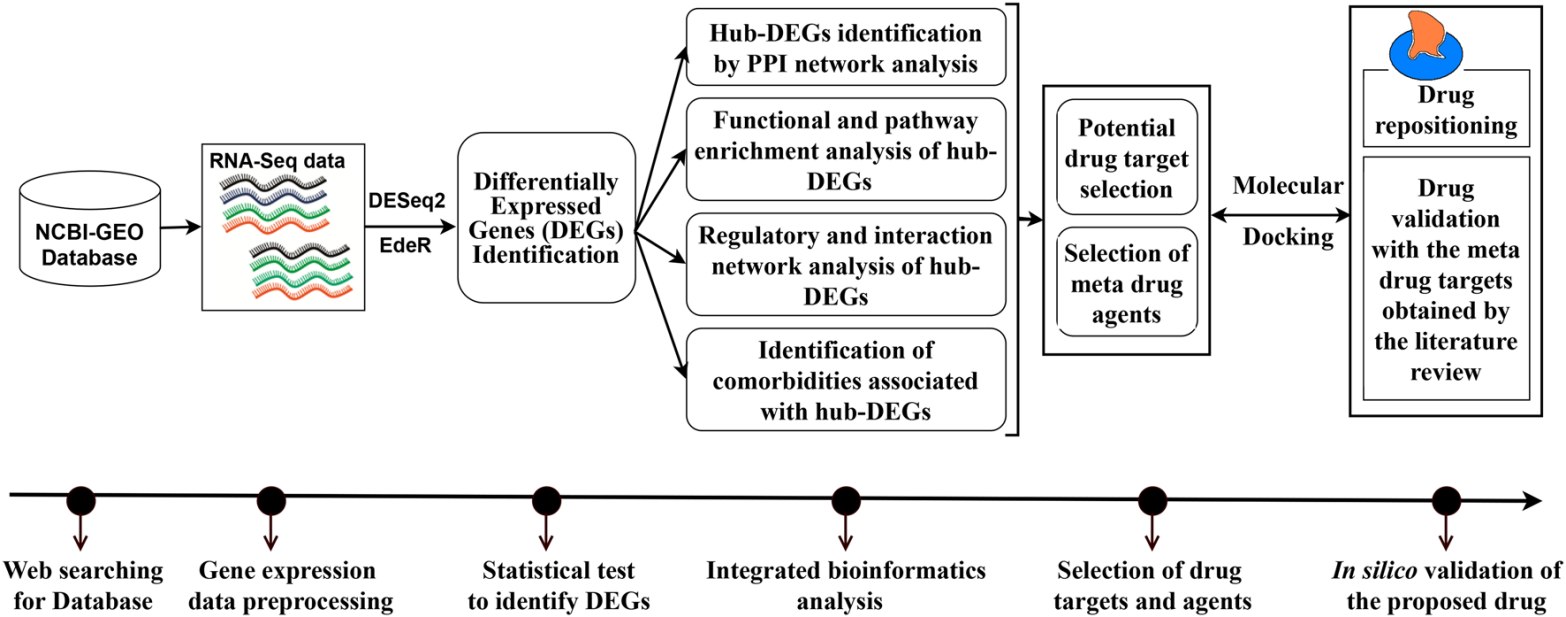
The pipeline of this study.

Though a number of vaccines including Pfizer, Moderna, Sputnik, AstraZeneca, Ad5-nCoV, EpiVacCorona, BBIBP-CorV, BBV152, CoronaVac, WIBP are now available against SARS-CoV-2 and some are in progress^16,17^, researchers are worried about their effectiveness due to unstable RNA sequence of coronaviruses. For example, recently we observed that some already vaccinated peoples are also suffering from SARS-CoV-2 infections in our surrounding. Therefore, besides the vaccines, different variants of well-established drugs are also required for the treatment against coronavirus to save human lives. However, new drug discovery is a tremendous challenging, time consuming and expensive task. The main challenges are to explore drug target receptor proteins responsible for diseases and drug candidate small molecules that can reduce the diseases by the interaction with the target proteins. Genomic biomarkers induced proteins are considered as the key drug target receptor proteins. Transcriptomics analysis is a widely used popular approach to explore genomic biomarkers^18–22^. The repurposing of existing drugs for a particular disease could reduce the time and cost compared to de novo drug development. By this time, several authors proposed host/SARS-CoV-2 transcriptome-guided several sets of candidate drugs by molecular docking with different sets of target proteins (receptors) for the treatment against SARS-CoV-2 infections^23–49^. However, their published data did not display any common set of receptors and/or drugs, and so far, none of them yet investigated the resistance of their suggested drugs against the independent receptors proposed by others. Therefore, how a vaccine or a drug can be effective for all the peoples around the world is still a question mark. In this study, our main objectives are (1) computational identification of genomic biomarkers (drug targets) for SARS-CoV-2 infections highlighting their functions, pathways, regulatory factors and associated comorbidities (2) exploring candidate drugs for the treatment against different variants of SARS-CoV-2 infections with comorbidities and (3) In-silico validation on the resistance of the proposed candidate drugs against the state-of-the-art alternatives top-ranked independent receptors associated with SARS-CoV-2 infections published by others. The overall analyses plan is summarized in Fig. 1 below.

## Results

### Identification of differentially expressed genes (DEGs)

**Figure 2:**
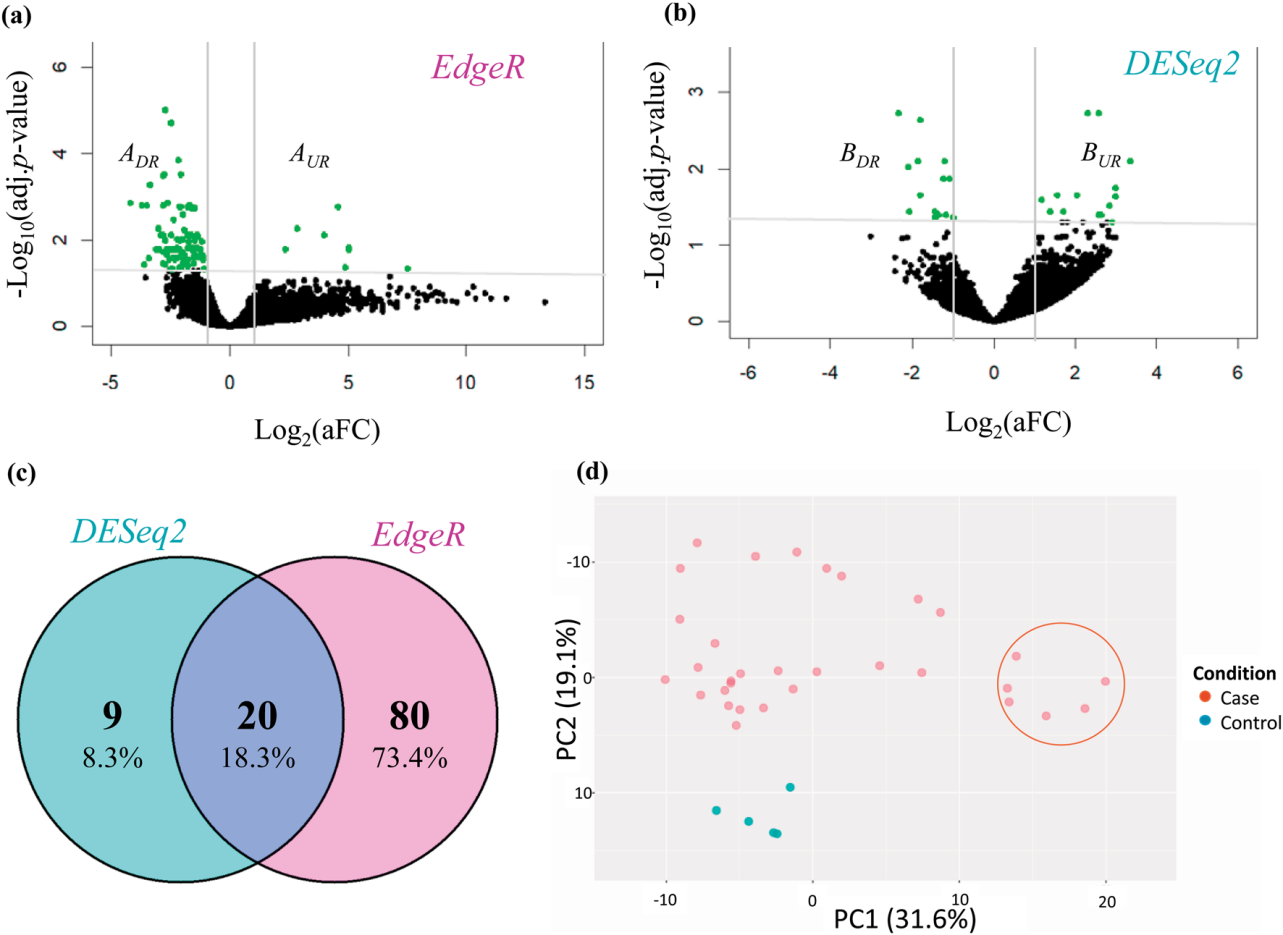
(**a**) Volcano plot of the DEGs by edgeR Method and (**b**) by DESeq2 method. (**c**) Venn diagram of two DEGs-sets identified by DESeq2 and edgeR to show the common and uncommon genes. (**d**) The scatter plot first two principal components (PCs) of DEGs to see their prognostic performance of the case vs control.

We identified the set of 109 DEGs as $({A}_{UR}\cup {A}_{DR})\cup ({B}_{UR}\cup {B}_{DR})$ between COVID-19 infected and control samples from RNA-Seq dataset by using two popular statistical R-packages (DESeq2 and edgeR) as introduced in the material and method section. The edgeR method identified the set of 100 DEGs as $({A}_{UR}\cup {A}_{DR})$, where 18 DEGs were upregulated (A_UR_) and the rest 82 DEGs were downregulated (A_DR_) (Fig. 2a). The DESeq2 method identified the set of 29 DEGs as $({B}_{UR}\cup {B}_{DR})$, where 15 DEGs were upregulated (B_UR_) and the rest 14 DEGs were downregulated (B_DR_) (Fig. 2b). The set of 20 DEGs was commonly identified by two methods as $\left({A}_{UR}\cup {B}_{UR}\right)\cap \left({A}_{DR}\cup {B}_{DR}\right)$, which is displayed by Venn diagram in Fig. 2c. Both the methods detected the DEGs with the significant level at adjusted *p*-value < 0.05 and the fold change threshold |log_2_(aFC_*g*_)|> 1 by controlling the FDR at 5%. We separated upregulated and downregulated DEGs by using fold change criteria log_2_(aFC_*g*_) > 1 and log_2_(aFC_*g*_) < − 1 respectively. However, we observed that a set of 2 DEGs denoted by C = {*ZNF638-IT1, FOSB}* showed contradictory results by edgeR and DESeq2. So, we removed these 2 DEGs from further analysis. Finally, we considered the set of 107 DEGs as $\left({A}_{UR}\cup {B}_{UR}\cup {A}_{DR}\cup {B}_{DR}\right)-C,$ which consisted of the set of 16 upregulated DEGs as ${DEG}_{UR}=\left({A}_{UR}\cup {B}_{UR}\right)-C$ and the set of 91 downregulated DEGs as ${DEG}_{DR}=\left({A}_{DR}\cup {B}_{DR}\right)-C$ (Table 2). To visualize sample clusters (case/control) and outliers simultaneously based on DEGs, we constructed the scatter plot of first and second principal components (PCs) of DEGs (Fig. 2d). We observed control samples are clearly separated from the case samples. So, the DEGs-set has a strong prognosis power. We also observed that 6 samples were contaminated by outliers (indicated with round line).

**Table 2:** The identified upregulated and downregulated DEGs-set.

Upregulated genes: ${DEG}_{UR}=\left({A}_{UR}\cup {B}_{UR}\right)\cap {C}^{/}$ (n = 16)	Downregulated genes: ${DEG}_{DR}=\left({A}_{DR}\cup {B}_{DR}\right)\cap {C}^{/}$ (n = 91)
*APLNR, CD79B, ITM2C, METTL24, PPP1R1A, RNU1-28P, RNU1-3, RP11-488L18.8, ATPIF1, FOLR2, H19, ICAM2, ****IGF2****, NDUFA8, PLVAP, ****SNRPD2***	*AC007278.2, AC007278.3, AC009303.1, AC068580.6, ACER3, AP001189.4, ARL5B, BEST1, CCL18, CCL20, CD300E, CTB89H12.4, CTD2537I9.18, ****CXCL2****, CYP1B1, CYR61, DESI2, DGKH, DUSP6, EDN1, ENPP4, ERAP2, GAPDHP59, GK, GLDN, ****GUCY1A2****, HS3ST2,IGFN1, IGSF10, ****KLF6****, LRRTM2, MTND1P23, NAMPTP1, NAV2-AS4, NAV2-AS5, NCR3LG1, ****NEDD9****, NLRP3, NUPL1, NUS1, OGFRL1, OSBPL8, ****PAG1****, PTP4A1, PZP, RASGEF1B, RNU2-61P, RP1-257A7.4, RP1-309I22.2, RP11-107E5.3, RP11-212I21.3, RP11-288K12.1, RP11-314N13.9, RP11-350J20.4, RP11-359E10.1, RP11-380G5.3, RP11-417F21.1, RP11-437B10.1, RP11-463O12.5, RP11-571F15.3, RP11-603J24.5, RP11-83B20.6, RP11-925D8.6, RP11-93B14.9, RP5-1101C3.1, RPL18P10, RRM2B, RSPO4, SDCBPP1, SDK2, SERPINB2, SLC16A6, SLC30A7, SLC5A8, SLC6A6, SLC7A11, SLC7A11-AS1, SLED1, SPDYA, SSXP10, TAF9BP1, TCF21, ****TLR2****, TRHDE, TSPAN19, TSPAN3, UPP1, ****USP53****, VGLL3, ZFAND5, ****ZFP36***
[The gene symbols in bold indicate hub DEGs that are obtained by PPI network analysis]	

### Protein–protein interaction (PPI) network analysis of DEGs

**Figure 3:**
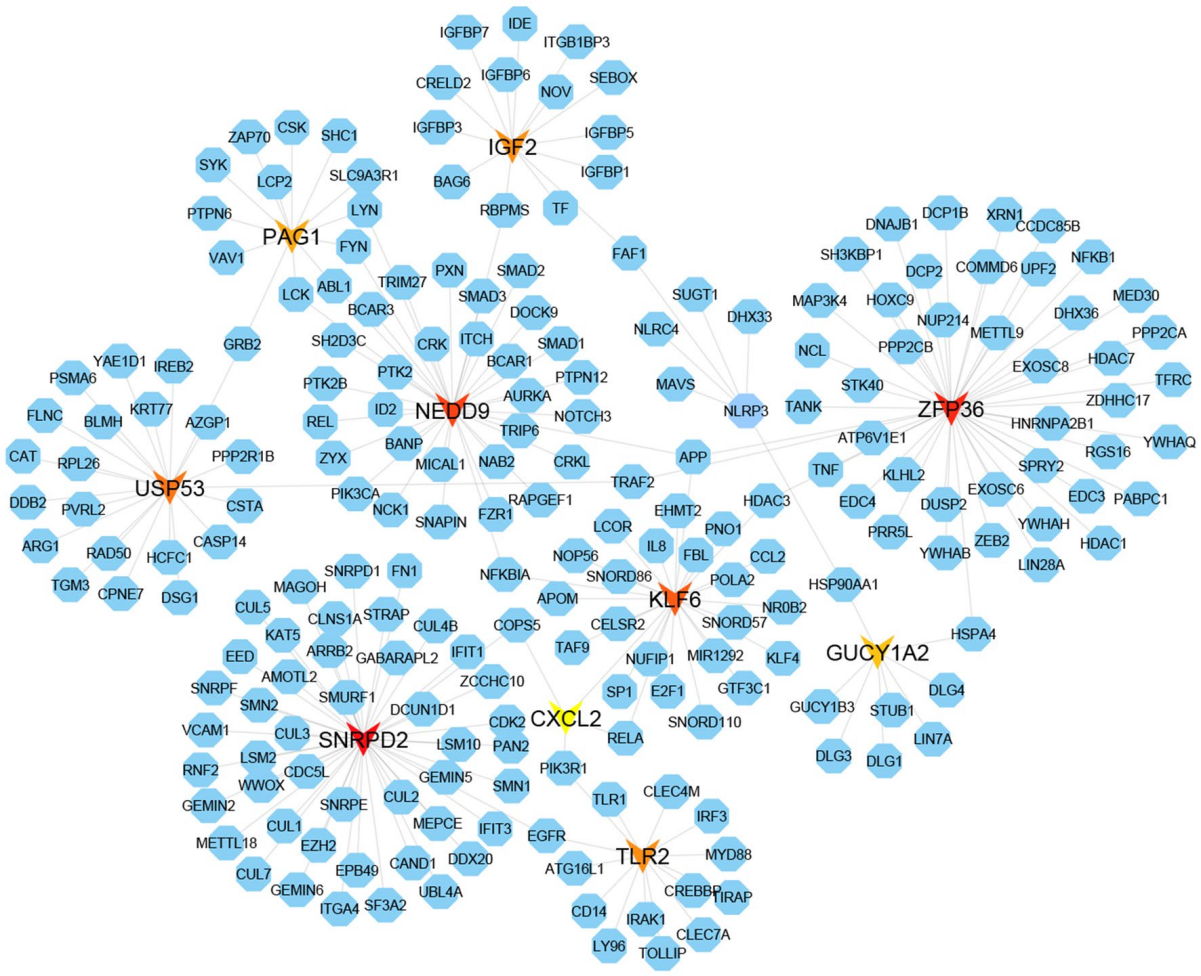
The PPI network of DEGs.

The PPI network of DEGs was constructed to detect the most representative DEGs so called hub-DEGs/proteins (see Fig. 3). A topological exploration based on dual-metric measurements (degree (> 10) and betweenness) was utilized to select the top-ranked 10 hub-DEGs/proteins that are SNRPD2, ZFP36, NEDD9, KLF6, USP53, IGF2, TLR2, PAG1, GUCY1A2 and CXCL2, where 2 hub-DEGs (IGF2 and SNRPD2) were upregulated and the remaining 8 were found as downregulated (see Table 2). These hub-DEGs/proteins were considered as the key controller of SARS-CoV-2 infections and drug target receptors.

### GO functional and KEGG pathway enrichment analysis of hub-DEGs

The GO functional enrichment analysis revealed that our proposed hub-DEGs are significantly enriched with numerous biological processes (BPs), molecular functions (MFs) and cellular components (CCs) (Table 3 and Supplementary File S1). The Table 3 shows top 10 significantly enriched GO-terms for each of three categories (MFs, BPs and CCs). We observed that 3 MFs (protein binding, binding, molecular function) were significantly enriched with all 10 hub-DEGs, 1 MF (protein-containing complex binding) was enriched with 3 hub-DEGs (IGF2, TLR2, GUCY1A2), 1 MF (C–C chemokine binding) was enriched with ZFP36 hub-DEG and the rest 5 MFs (triacyl lipopeptide binding, lipopolysaccharide immune receptor activity, lipopeptide binding, NAD+ nucleotidase, cyclic ADP-ribose generating and NAD+ nucleosidase activity) were significantly enriched with TLR2. Out of top 10 significantly enriched GO-terms of BPs by hub-DEGs, we observed that 2 BPs (biological process and cellular process) were highly enriched by all of 10 hub-DEGs, 5 BPs, (biological regulation, response to stimulus, positive regulation of cellular process, signal transduction and immune system process) were enriched by the individual sub DEGs-sets including 9, 8, 7, 7 and 6 hub-DEGs, respectively. The other 3 GO-terms (cellular response to lipopolysaccharide, cellular response to molecule of bacterial origin and cellular response to biotic stimulus) of BPs were enriched by the same 3 hub-DEGs (ZFP36, TLR2, CXCL2). Among the top 10 significantly enriched GO-terms of CCs by hub-DEGs, 2 CCs (cellular component and cellular anatomical entity) were enriched by all 10 hub-DEGS, 7 hub-DEGs significantly enriched with the cytoplasm of CCs, 2 hub-DEGs (TLR2 and PAG1) significantly enriched with 3 GO-terms membrane raft, membrane microdomain and membrane region of CCs and the rest 4 CCs (Dcp1-Dcp2 complex, Toll-like receptor 1-Toll-like receptor 2 protein complex, pICln-Sm protein complex and RISC-loading complex) were enriched by ZFP36, TLR2, SNRPD2 and ZFP36, respectively.

**Table 3:** The top 10 significantly enriched GO terms for each of BPs, MFs and CCs with the hub-DEGs/proteins.

Source	GO term name	GO term id	Adj. p-value	Count	Associated hub DEGs or proteins
GO: MF	Protein binding	GO:0005515	0.0000	10	SNRPD2, ZFP36, NEDD9, KLF6, USP53, IGF2, **TLR2**, PAG1, GUCY1A2, CXCL2
	Binding	GO:0005488	0.0001	10	SNRPD2, ZFP36, NEDD9, KLF6, USP53, IGF2, **TLR2**, PAG1, GUCY1A2, CXCL2
	Molecular function	GO:0003674	0.0002	10	SNRPD2, ZFP36, NEDD9, KLF6, USP53, IGF2, **TLR2, **PAG1, GUCY1A2, CXCL2
	Triacyl lipopeptide binding	GO:0042497	0.0049	1	**TLR2**
	Protein-containing complex binding	GO:0044877	0.0185	3	IGF2, **TLR2**, GUCY1A2
	Lipopolysaccharide immune receptor activity	GO:0001875	0.0185	1	**TLR2**
	C–C chemokine binding	GO:0019957	0.0194	1	ZFP36
	Lipopeptide binding	GO:0071723	0.0194	1	**TLR2**
	NAD+ nucleotidase, cyclic ADP-ribose generating	GO:0061809	0.0194	1	**TLR2**
	NAD+ nucleosidase activity	GO:0003953	0.0194	1	**TLR2**
GO: BP	Cellular response to lipopolysaccharide	GO:0071222	0.0009	3	ZFP36, **TLR2**, CXCL2
	Immune system process	GO:0002376	0.0009	6	ZFP36, KLF6, IGF2, **TLR2**, PAG1, CXCL2
	Positive regulation of cellular process	GO:0048522	0.0009	7	ZFP36, NEDD9, KLF6, IGF2, **TLR2**, PAG1, GUCY1A2
	Biological regulation	GO:0065007	0.0009	9	ZFP36, NEDD9, KLF6, USP53, IGF2, **TLR2**, PAG1, GUCY1A2, CXCL2
	Biological_process	GO:0008150	0.0009	10	SNRPD2, ZFP36, NEDD9, KLF6, USP53, IGF2, **TLR2**, PAG1, GUCY1A2, CXCL2
	Cellular process	GO:0009987	0.0009	10	SNRPD2, ZFP36, NEDD9, KLF6, USP53, IGF2, **TLR2**, PAG1, GUCY1A2, CXCL2
	Cellular response to molecule of bacterial origin	GO:0071219	0.0009	3	ZFP36, **TLR2**, CXCL2
	Cellular response to biotic stimulus	GO:0071216	0.0010	3	ZFP36, **TLR2,** CXCL2
	Signal transduction	GO:0007165	0.0011	7	ZFP36, NEDD9, IGF2, **TLR2**, PAG1, GUCY1A2, CXCL2
	Response to stimulus	GO:0050896	0.0011	8	ZFP36, NEDD9, USP53, IGF2, **TLR2**, PAG1, GUCY1A2, CXCL2
GO: CC	Cellular_component	GO:0005575	0.0005	10	SNRPD2, ZFP36, NEDD9, KLF6, USP53, IGF2, **TLR2**, PAG1, GUCY1A2, CXCL2
	Cellular anatomical entity	GO:0110165	0.0005	10	SNRPD2, ZFP36, NEDD9, KLF6, USP53, IGF2, **TLR2**, PAG1, GUCY1A2, CXCL2
	Dcp1–Dcp2 complex	GO:0098745	0.0066	1	ZFP36
	Toll-like receptor 1-Toll-like receptor 2 protein complex	GO:0035354	0.0099	1	**TLR2**
	cytoplasm	GO:0005737	0.0161	7	SNRPD2, ZFP36, NEDD9, KLF6, IGF2, **TLR2**, GUCY1A2
	pICln-Sm protein complex	GO:0034715	0.0178	1	SNRPD2
	Membrane raft	GO:0045121	0.0178	2	**TLR2**, PAG1
	Membrane microdomain	GO:0098857	0.0178	2	**TLR2**, PAG1
	RISC-loading complex	GO:0070578	0.0178	1	ZFP36
	Membrane region	GO:0098589	0.0178	2	**TLR2**, PAG1

**Figure 4:**
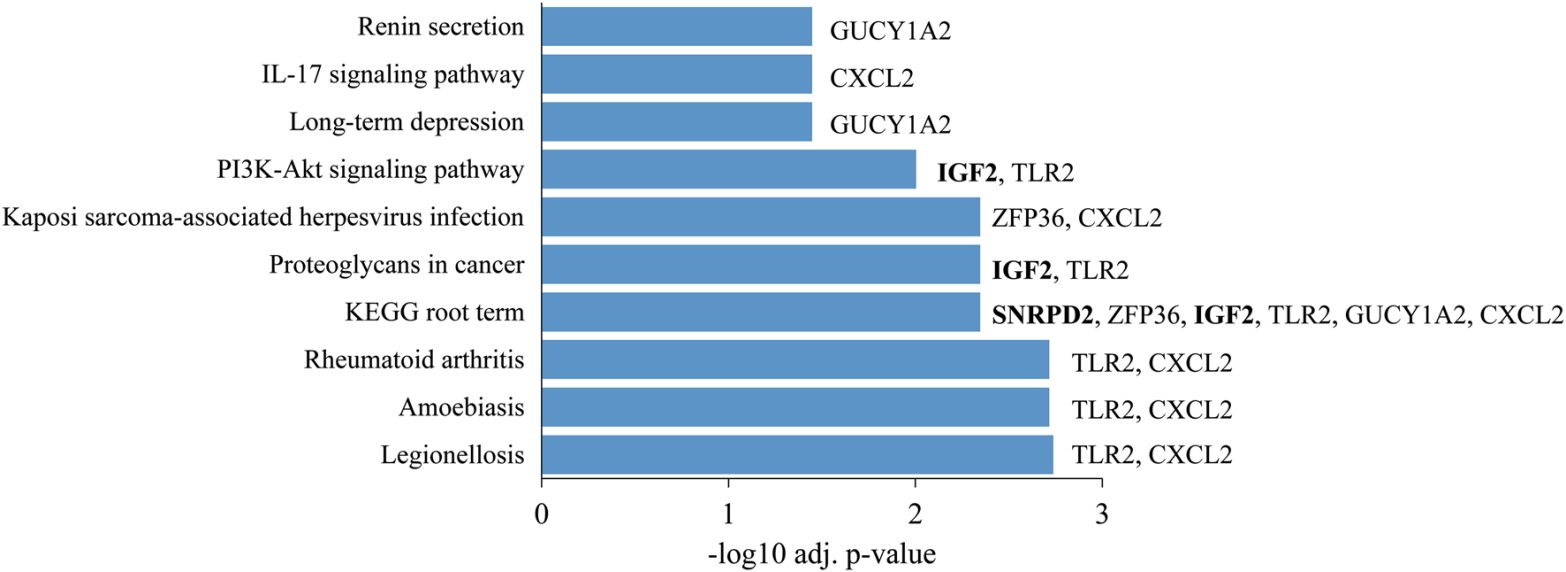
The top 10 significantly enriched KEGG pathways with the hub-DEGs/proteins. The associated hub-DEGs are displayed in the right side of each bar. The hub-DEGs with bold represents upregulated genes and others are downregulated genes.

The KEGG pathway enrichment analysis exposed that our proposed hub-DEGs are significantly enriched with plentiful pathway categories (see Fig. 4 and Supplementary File S1). The Fig. 4 showed the top 10 significantly enriched KEGG-pathway categories. We observed that the KEGG root term is significantly enriched with 7 hub-DEGs (SNRPD2, ZFP36, IGF2, TLR2, GUCY1A2, and CXCL2). Each of three pathway categories legionellosis, amoebiasis and rheumatoid arthritis was enriched by two hub-DEGs (TLR2 and CXCL2). Each of two pathway categories (proteoglycans in cancer and PI3K-Akt signaling) was enriched by two hub-DEGs (TLR2 and IGF2). The pathway category Kaposi sarcoma-associated herpesvirus infection was enriched by two hub-DEGs (ZFP36 and CXCL2). The two KEGG pathway categories (long-term depression and renin secretion) was enriched by the hub-DEG GUCY1A2. The rest one pathway category IL-17 signaling pathway was enriched by the hub-DEG CXCL2.

### The gene regulatory network (GRN) analysis of hub-DEGs

**Figure 5:**
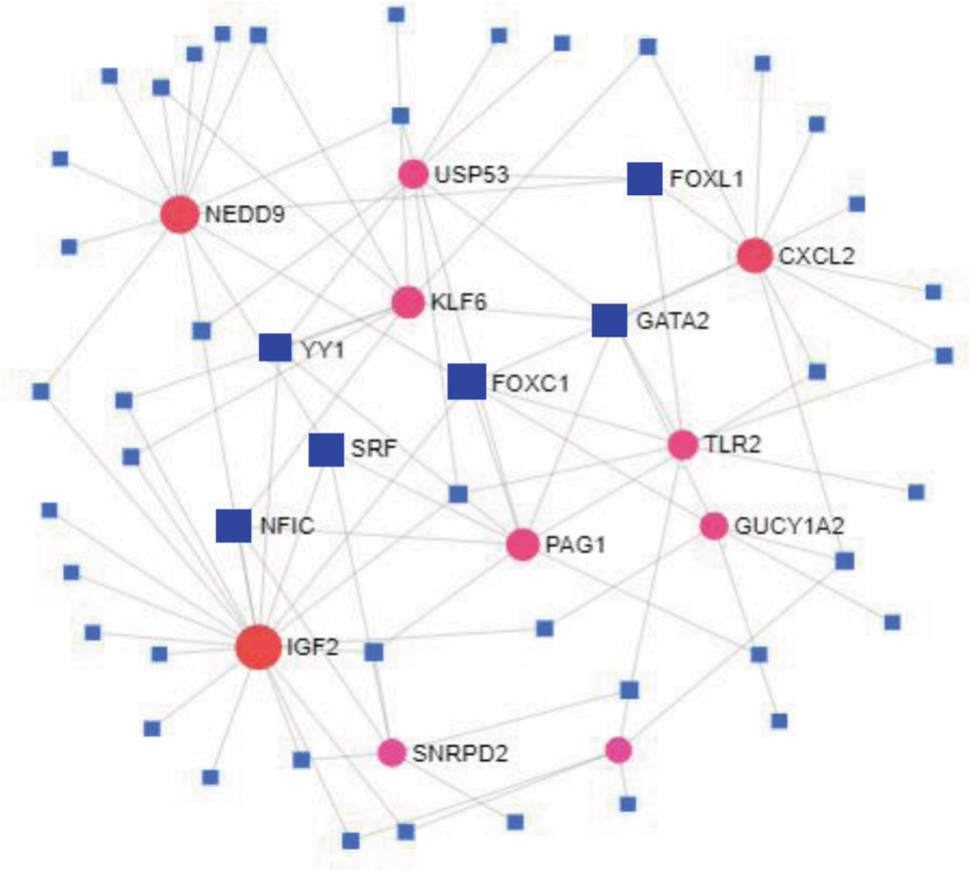
The TFs and hub-DEGs interaction network, where the blue squared nodes represent the TFs and the red round nodes represent the hub-DEGs. The key TFs are represented by the larger squared nodes.

Transcription factors (TFs) and microRNAs (miRNAs) are considered as the most important transcriptional and post transcriptional molecular regulatory factors of genes. We constructed undirected interaction network between TFs and hub-DEGs to explore key regulatory transcriptional factors of hub-DEGs (Fig. 5). In this network, hub-DEGs were represented by round nodes with red color and TFs were represented by square nodes with blue color, where larger numbers of connectivity were represented by the larger nodes. We selected FOXC1, GATA2, SRF, FOXL1, YY1 and NFIC as the top 6 key regulatory TFs of hub-DEGs based on higher degree of topological measures.

**Figure 6:**
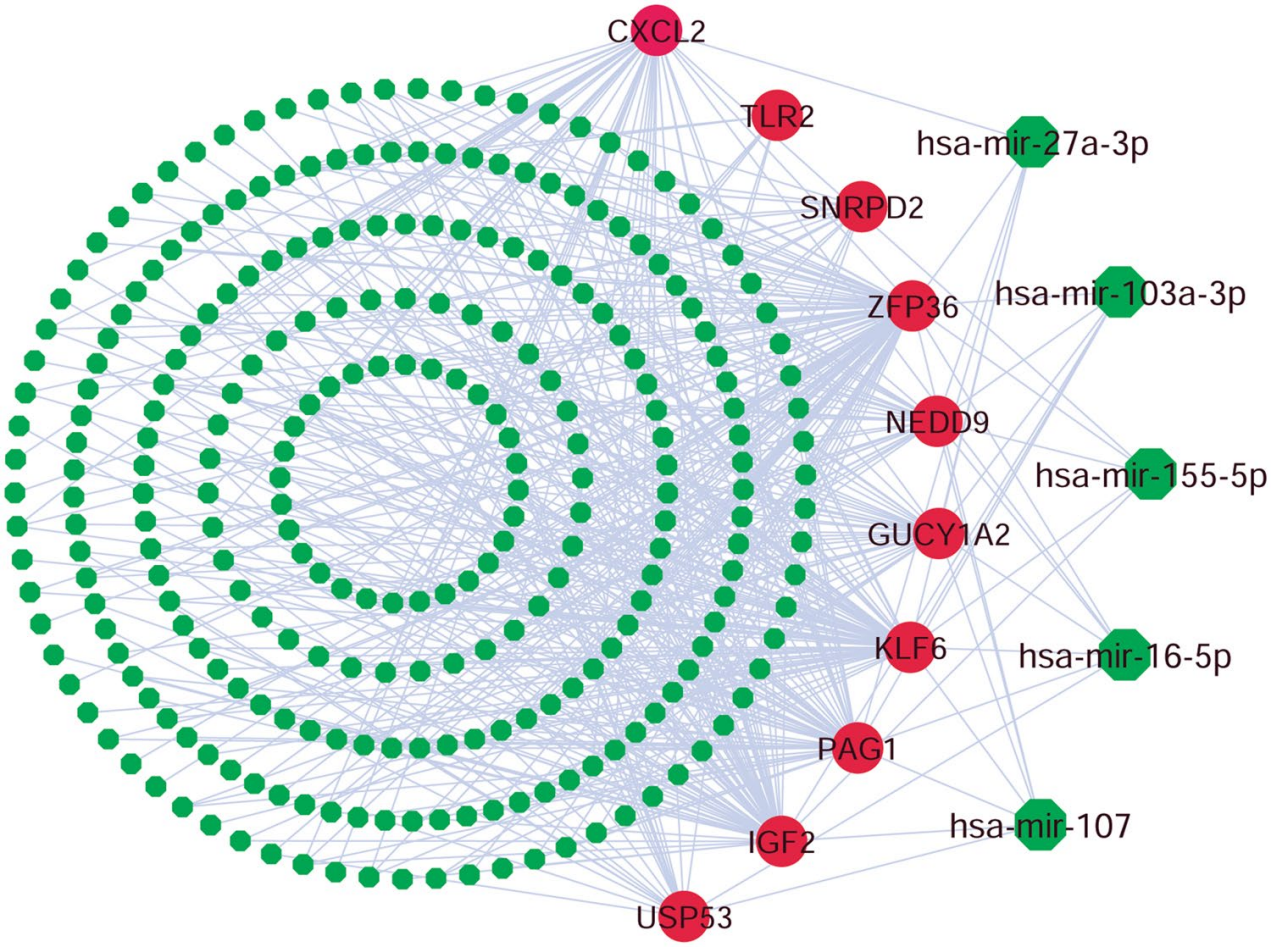
The miRNAs and hub-DEGs interaction network where the small green octagonal nodes stand for miRNAs and the round nodes with red color represents the hub-DEGs. The key miRNAs are represented by the larger highlighted octagonal shaped green colored nodes in the figure.

To explore key regulatory post-transcriptional factors of hub-DEGs, we constructed undirected interaction network between miRNAs and hub-DEGs (Fig. 6). In this network, hub-DEGs were represented by round nodes with red color and miRNAs were represented by octagonal nodes with green color, where larger numbers of connectivity were represented by the larger nodes as before. We selected the miRNAs namely, miR-107, miR-16-5p, miR-103a-3p, miR-27a-3p, miR-155-5p and miR-1-3p as the top 6 key regulatory post-transcriptional factors of hub-DEGs based on higher degree of topological measures.

### Association of hub-DEGs with comorbidities

**Figure 7:**
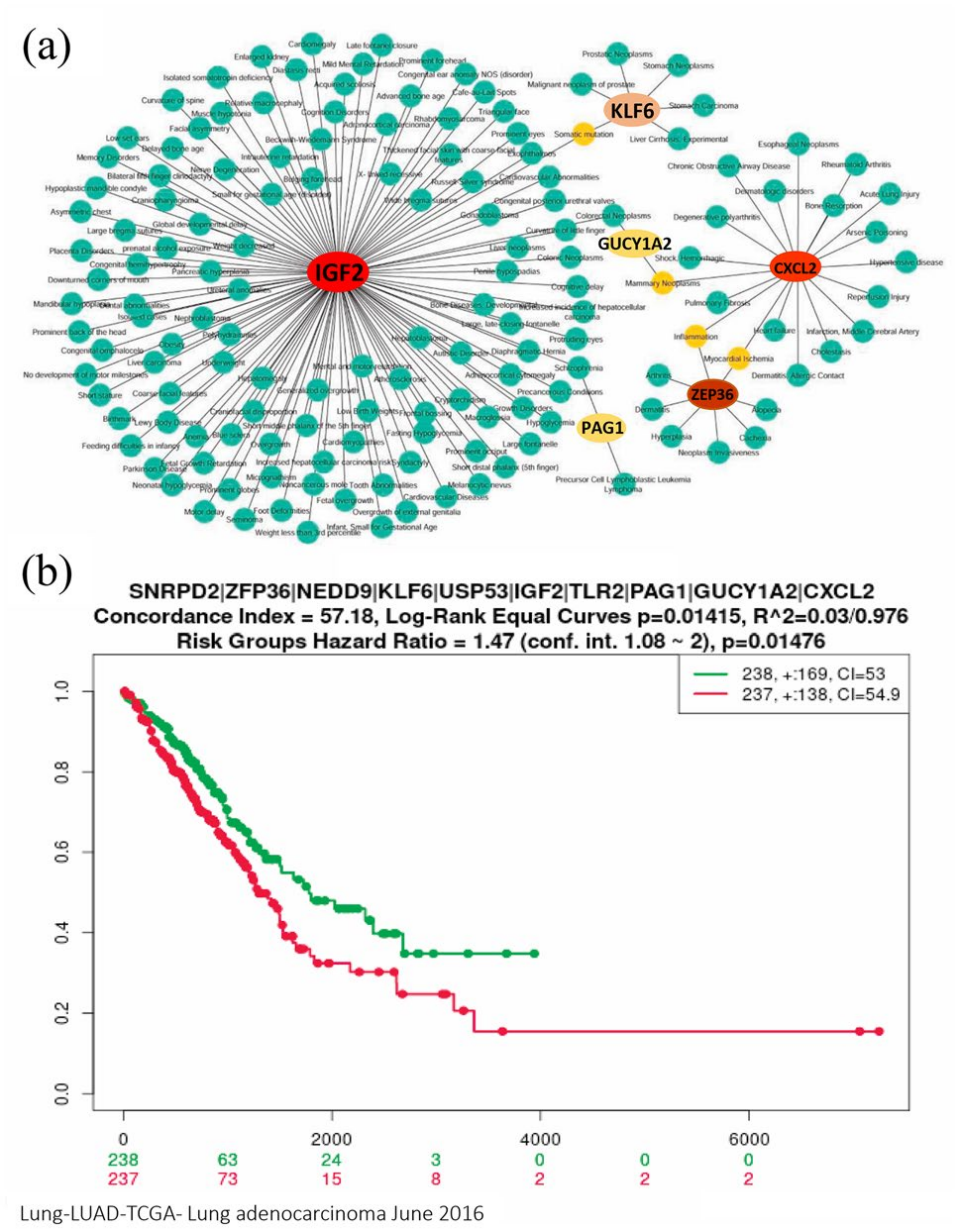
(**a**) The disease versus hub-DEGs interaction network finds the comorbidities (**b**) The multivariate survival curves of lung cancer patients based on hub-DEGs.

To investigate the association of hub-DEGs with other diseases, we performed their interaction network analysis. The Fig. 7a and Supplementary Table S2 show the disease versus hub-DEGs interaction network analysis results. We observed that IGF2 gene is associated with 120 diseases including Cardiovascular Diseases, Colorectal Neoplasms, Cardiomyopathies, Liver carcinoma, Anemia; the CXCL2 gene is associated with 19 diseases including rheumatoid arthritis, heart failure, hypertensive disease, IBN inflammation, pulmonary fibrosis, acute lung injury while the ZFP36, KLF6, GUCY1A2 and PAG1 was linked with 18 diseases including liver cirrhosis, experimental prostatic neoplasms, stomach carcinoma, inflammation, arthritis, especially which could be a severe comorbidities of the COVID-19 patients. To assess the association of hub-DEGs with lung cancer, we performed multivariate survival analysis of lung cancer patients with expressions of hub-DEGs. The log-rank test was used to test the significant difference between two survivals curves (Fig. 7b) based on all hub-DEGs. We observed the significant difference between the low and high-risk group in survival probability, which indicates that the hub proteins are significantly associated with lung cancer.

### Drug repurposing by molecular docking study

**Figure 8:**
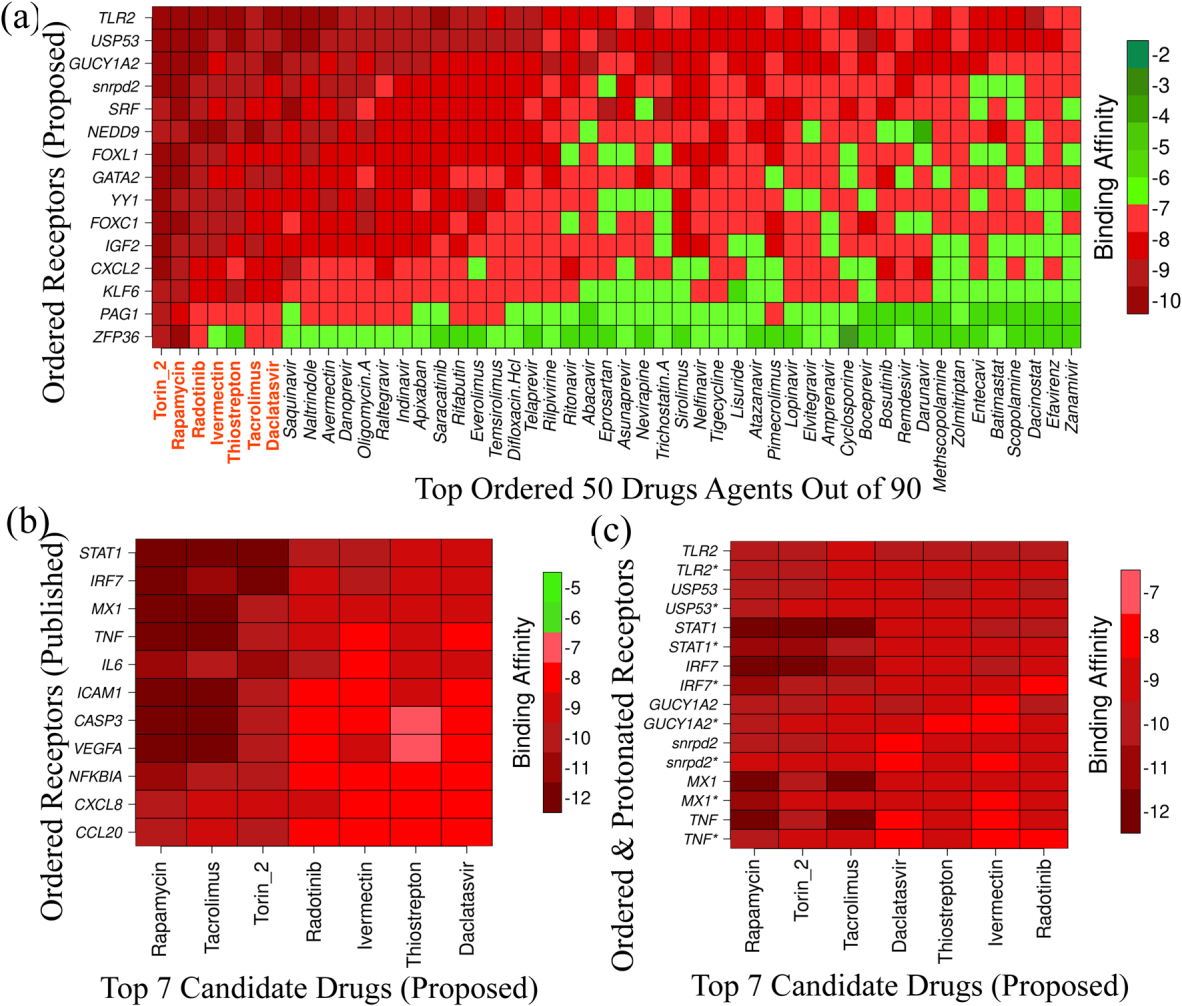
Molecular docking results computed with autodock vina. Red colors indicated the strong binding affinities between target proteins and drug agents, and green colors indicated their weak bindings. (**a**) Image of binding affinities based on the top 50 ordered drug agents out of 90 in X-axis and ordered 15 target proteins (proposed) in Y-axis. (**b**) Image of binding affinities based on the proposed ordered 7 candidate drugs in X-axis and ordered 11 independent receptors (already published) in Y-axis. (**c**) Image of binding affinities based on the proposed ordered 7 candidate drugs in X-axis and original & protonated (at pH-7)* receptors in Y-axis, where * indicates the protonated receptors.

We considered 10 hub-proteins corresponding to our proposed 10 hub-DEGs and their regulatory 5 key TFs-proteins (FOXC1, GATA2, SRF, FOXL1 and YY1) as the *m* = 15 drug target receptors. The SARS-CoV-2 3CL protease-guided top-ranked *n* = 90 drugs out of 3410 FDA approved antiviral drugs^50^, were considered as the drug agents that were mentioned previously in the materials and method section. We downloaded 3D structure of 7 hub proteins (SNRPD2, ZFP36, NEDD9, IGF2, TLR2, and CXCL2) from Protein Data Bank (PDB)^51^ with source codes 5xjs, 4j8s, 2l81, 1igl, 1fyw, and 5ob5, respectively. The 3D structure of 3 hub proteins (KLF6, USP53, GUCY1A2, and PAG1) were downloaded from SWISS-MODEL^52^ using UniProt^53^ ID of Q99612, Q70EK8, P33402, and Q9NWQ8, respectively. The 3D structure of 3 TFs proteins (GATA2, SRF and YY1) were downloaded from PDB with source codes 5o9b, 1hbx, and 1ubd, respectively, and the rest 2 TFs proteins (FOXC1 and FOXL1) were downloaded from SWISS-MODEL using UniProt ID of Q12948 and Q12952. The 3D structures of 90 FDA-approved drugs were downloaded from PubChem database^54^. Then we performed molecular docking study to calculate binding affinity scores for each pair of receptors and drug agents. Then we ordered the receptor proteins according to the descending order of row sums of the binding affinity score matrix ***A*** = (*A*_ij_) and drug agents according to the column sums to select the top-ranked few drug agents as the candidate drugs. Figure 8a displayed the image of binding affinity matrix ${{\varvec{A}}}^{*}=\left({A}_{ij}^{*}\right)$ corresponding to the ordered target proteins in Y-axis and top-ordered 50 drug agents out of 90 in X-axis. We observed that the first two top lead compounds (lead1: Torin-2 and lead2: Rapamycin) produce binding affinity scores less than or equal to − 8.0 kcal/mol with all of our proposed 15 receptor proteins. The next (3–7)th top lead compounds (lead3:Radotinib, lead4:Ivermectin, lead5:Thiostrepton, lead6:Tacrolimus and lead7:Daclatasvi) produced binding affinity scores less than or equal to − 8.0 kcal/mol with our proposed 13 receptor proteins out of 15. The rest 83 lead compounds produced binding affinity scores less than or equal to − 8.0 kcal/mol with the fewer number of receptors. Therefore, we considered the top 7 lead compounds (Torin-2, Rapamycin, Radotinib, Ivermectin, Thiostrepton, Tacrolimus and Daclatasvi) as the most probable candidate drugs for SARS-CoV-2 infections.

To validate our proposed 7 candidate drugs by molecular docking study with already published independent receptor proteins (genomic biomarkers) associated with SARS-CoV-2 infections available in the literature, we reviewed 22 published articles associated with SARS-CoV-2 infections those provided transcriptome guided hub-proteins (genomic biomarkers). We found total of 193 hub-proteins published in those 22 articles, but there was no hub-protein commonly published in all articles (Table 4). We found 11 hub-proteins (ICAM1, IRF7, MX1, NFKBIA, STAT1, IL6, TNF, CCL20, CXCL8, VEGFA, and CASP3) which were commonly reported in at most 3 articles out of 22 (Table 4). We considered these 11 hub-proteins as the publicly available top ranked receptor proteins associated with SARS-CoV-2 infections to validate the proposed repurposed drugs by molecular docking. The 3D structures of these 11 (ICAM1, IRF7, MX1, NFKBIA, STAT1, IL6, TNF, CCL20, CXCL8, VEGFA, and CASP3) proteins were retrieved from Protein Data Bank (PDB) with codes 5mza, 2o61, 3szr, 1nfi, 1bf5, 1il6, 1tnf, 2jyo, 1ikl, 1cz8, and 1gfw, respectively. Then molecular docking interactions of our proposed drugs with the publicly available top ranked receptor proteins were performed. Their binding affinities (kcal/mol) were visualized in Fig. 8b. We observed that their binding affinities ranged between (− 12.1 to − 7) kcal/mol and average binding affinities were less than or equal to − 9.5 kcal/mol which indicates the strong binding capacity. Then we compared the docking results of top-ranked eight receptor proteins (four from the proposed set and the other four from the published set) with their protonation state at their physical conditions of salinity = 0.15, internal dielectric = 10, pH = 7, and external dielectric = 80 (see Supplementary File S4)^55^. The docking analysis showed the significant binding affinities ranged between (− 11 to − 7.7) kcal/mol with the protonated receptors (Fig. 8c). We observed that both original and protonated (*) receptors produce almost similar binding scores, which indicate the proposed drugs might be effective against the protonated receptors also.

**Table 4:** Different key protein lists for SARS-CoV-2 infection published by different research articles in different international reputed journals.

Articles	Hub-proteins	Common hub genes with at least 3 articles	Common hub genes with at least 4 articles
Xie et al.^45^	CXCL1, CXCL2, TNF, NFKBIA, CSF2, TNFAIP3, IL6, CXCL3, CCL20, ICAM1	IL6, TNF, CCL20, CXCL8, VEGFA, ICAM1, IRF7, MX1, NFKBIA, STAT1, CASP3	IL6, TNF, VEGFA
Oh et al.^46^	GATA4, ID2, MAFA, NOX4, PTBP1, SMAD3, TUBB1, WWOX		
Vastrad et al.^47^	TP53, HRAS, CTNNB1, FYN, ABL1, STAT3, STAT1, JAK2, C1QBP, XBP1, BST2, CD99, IFI35, MAPK11, RELA, LCK, KIT, EGR1, IL20, ILF3, CASP3, IL19, ATG7, GPI, S1PR1		
Prasad et al.^48^	STAT1, IRF7, IFIH1, MX1, ISG15, IFIT3, OAS2, DDX58, IRF9, IFIT1, OAS1, OAS3, DDX60, OASL, IFIT2		
Selvaraj et al.^49^	MYC, HDAC9, NCOA3, CEBPB, VEGFA, BCL3, SMAD3, SMURF1, KLHL12, CBL, ERBB4, CRKL		
Satu et al.^28^	MARCO, VCAN, ACTB, LGALS1, HMOX1, TIMP1, OAS2, GAPDH, MSH3, FN1, NPC2, JUND, GPNMB, SYTL2, CASP1, S100A8, MYO10, IGFBP3, APCDD1, COL6A3, FABP5, PRDX3, CLEC1B, DDIT4, CXCL10, CXCL8		
Taz et al.^29^	VEGFA, AKT1, MMP9, ICAM1, CD44		
Moni et al.^30^	MX1, IRF7, BST2		
Islam et al.^31^	BIRC3, ICAM1, IRAK2, MAP3K8, S100A8, SOCS3, STAT5A, TNF, TNFAIP3, TNIP1		
Zhou et al.^32^	JUN, XPO1, NPM1, HNRNPA1		
Ge et al.^33^	MMP13, NLRP3, GBP1, ADORA2A, PTAFR, TNF, MLNR, IL1B, NFKBIA, ADRB2, IL6		
Aishwarya et al.^34^	IGF2, HINT1, MAPK10, SGCE, HDAC5, SGCA, SGCB, CFD, ITSN1, EHMT2, CLU, ISLR, PGM5, ANK2, HDAC9, SYT11, MDH1, SCCPDH, SIRT6, DTNA, FN1, ARRB1, MAGED2, TEX264, VEGFC, HK2, TXNL4A, SLC16A3, NUDT21, TRA2B, HNRNPA1, CDC40, THOC1, PFKFB3		
Saxena et al.^35^	STAP1, CASP5, FDCSP, CARD17, ST20, AKR1B10, CLC, KCNJ2-AS1, RNASE2, FLG		
Tao et al.^36^	MAPK3, MAPK8, TP53, CASP3, IL6, TNF, MAPK1, CCL2, PTGS2		
Zhang et al.^37^	CXCL10, ISG15, DDX58, MX2, OASL, STAT1, RSAD2, MX1, IRF7, OAS1		
Han et al.^38^	IL6, TNF,IL10, MAPK8,MAPK3,CXCL8,CASP3,PTGS2, TP53, MAPK1		
Wang et al.^39^	CXCL8, CXCL1, CXCL2, CCL20, CSF2		
Gu et al.^40^	NFKBIA, C3, CCL20, BCL2A1, BID		
Nan et al.^41^	ALB, CXCL8, FGF2, IL6, INS, MMP2, MMP9, PTGS2, STAT3, VEGFA		
Gu et al.^42^	CDC20, NCBP1, POLR2D, DYNLL1, FBXW5, LRRC41, FBXO21, FBXW9, FBXO44, FBXO6		
Sardar et al.^43^	HMOX1, DNMT1, PLAT, GDF1, ITGB1		
Gu et al.^44^	FLOC, DYNLL1, FBXL3, FBXW11, FBXO27, FBXO44, FBXO32, FBXO31, FBXO9, CUL2		

Table 5, Supplementary Files S3 and S4 show the summary results of interacting properties of our target proteins with top-ranked drugs (lead compounds) that produced the highest binding affinity scores. The 3D structure of their interacting complex is shown in the 4th column of Table 5. The 2D schematic diagram of these 3 target proteins with mentioned candidate drugs interaction is given in the 5th column highlighting their neighbor residues (within 4 Å of the drug). Key interactions amino acids and their binding with potential targets were shown in the last column.

**Table 5:** Top 3 potential targets and top 3 lead compounds based on docking results. Lead three compounds Torin-2, Rapamycin, and Radotinib were selected by investigating the binding affinity score. The 3D structure of hub protein with candidate drugs is shown in 4th column. The 2D Schematic diagram of hub protein with candidate drugs interaction is given 5th column and neighbor residues (within 4 Å of the drug) are shown. Key interactions amino acids and their binding with potential targets were shown in the last column.

Potential targets	Structure of lead compounds	Binding affinity # (kCal/mol)	The 3d view of complex	Ligand interactions	Interacting amino acids			
					Hydrogen bond	Hydrophobic interactions	Halogen/salt bridge	π-Stacking
TLR2		− 9.8			SER696, HIS721	GLU694, PHE722, ARG723, LEU724	–	PHE722
TLR2		− 9.7			Lys754	Phe701, Trp712, Asp726, Glu727, Ala732, Leu734, Lys751, Ile755, Thr758, Thr760	–	–
USP53		− 9.5			ARG199, ASP219, ARG236	TYR183, PHE208, THR218	ILE234	PHE208

### Molecular dynamic (MD) simulations

Among the proposed candidate drugs, Torin-2, Rapamycin and Radotinib were the top ranked three candidate drugs (Table 5). Therefore, these top three drug agents were selected for their stability analysis through 100 ns MD-based MM-PBSA simulations.

### RMSD analysis

**Figure 9:**
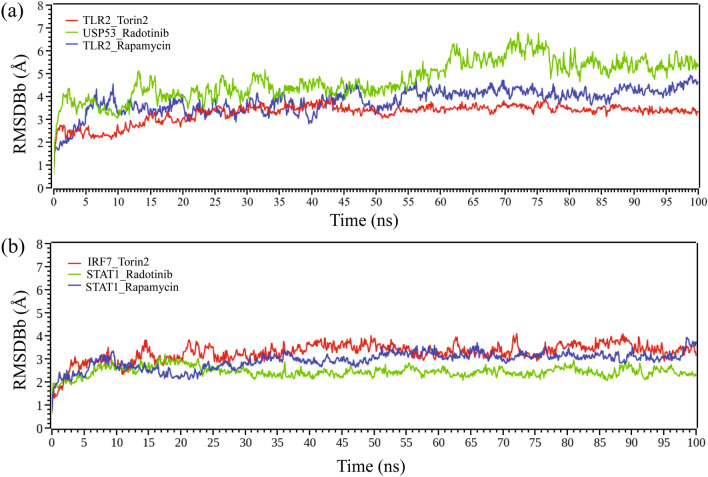
The RMSD analysis results for the variations of moving and initial drug-target complexes with 100 ns based MD simulations. (**a**) represented the RMSD with proposed top ranked two receptors (TLR2, USP53) and (**b**) represented the RMSD with top ranked two independent receptors (IRF7, STAT1).

From Fig. 9, we observed that all the six systems are significantly stable between the variations of moving and initial drug-target complexes. We calculated their RMSD (root mean square deviation). Figures 9a,b, represents the RMSD corresponding to the proposed receptors (TLR2, USP53) and independent receptors (IRF7, STAT1), respectively. All the systems projected the RMSD around 2 to 4 Å except USP53_Radotinib complex which showed the RMSD around 3.5 to 6.5 Å. The average RMSD for TLR2_Torin-2, TLR2_Rapamycin and USP53_Radotinib complexes were 3.3 Å, 3.7 Å and 4.7 Å, respectively. TLR2_Torin-2 complex showed slight fluctuation in Cα backbone around 40,000 ps and was stabilized in the remaining simulation. Similarly, a streak of continuous fluctuation was found in the TLR2_Rapamycin complex, ranging from 30,000 to 56,000 ps, followed by inconsiderable change. For USP53_radotinib complex, a negligible fluctuation was observed in the starting 12,000 ps and around 62,000 ps to 80,000 ps and remained stable thereafter throughout the simulation. On the other hand, the average RMSD was found to be 3.3 Å for IRF7_Torin-2 complex with slight fluctuation in Cα backbone around 12,000 to 16,000 ps and was stabilized in the remaining simulation. For STAT1_Rapamycin complex, the average RMSD was found to be 2.9 Å. The average RMSD for the STAT1_radotinib bound complex was 2.4 Å, with an overall RMSD of approximately 2.6 Å, indicating that it was comparably more stable among the six selected systems. However, the data indicates that all the systems showed stable internal motion.

### Binding free energy

**Figure 10:**
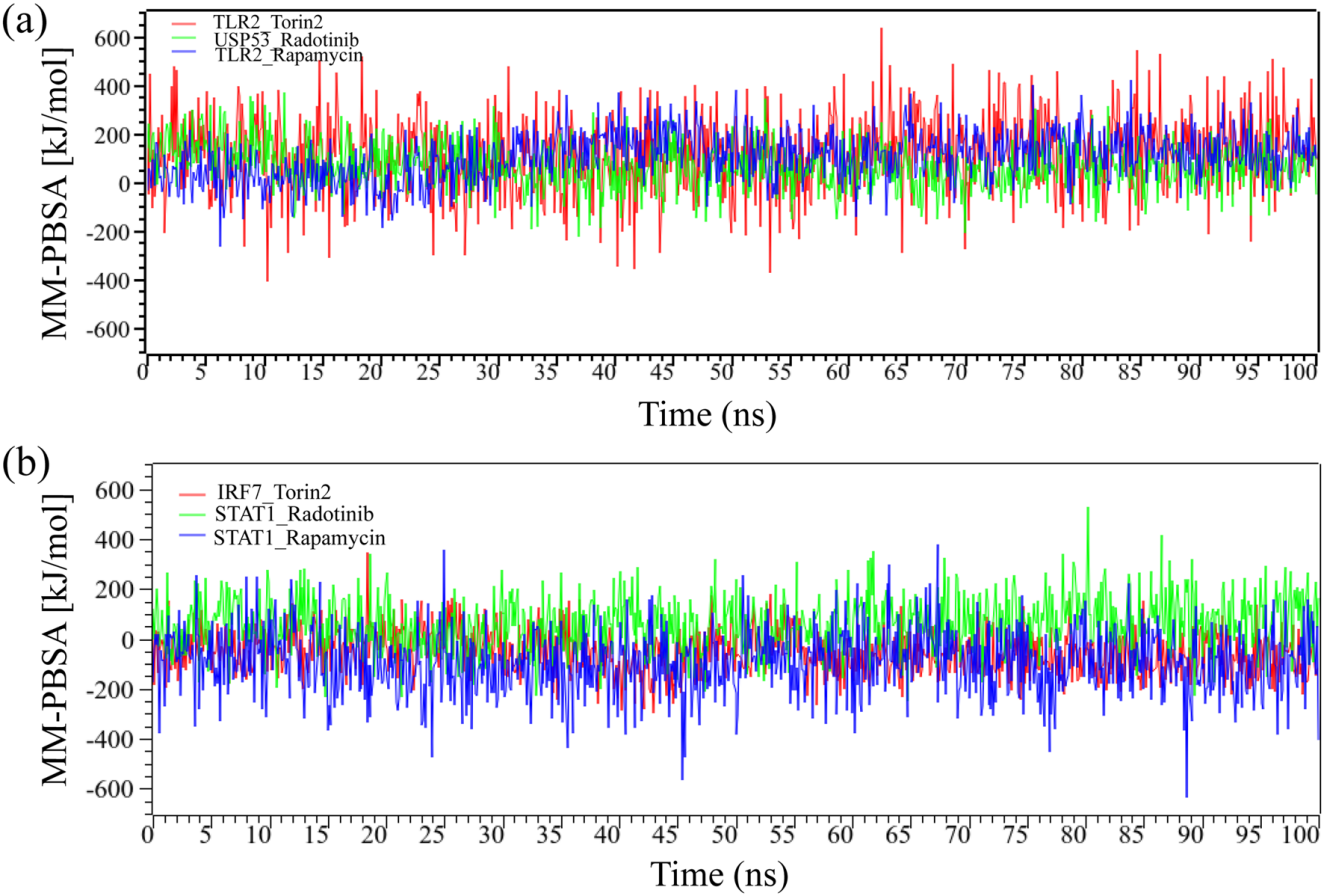
Binding free energy (in kJ mol^−1^) of each snapshot was calculated through 100 ns MD-based MM-PBSA simulations (**a**) represented the binding free energy with proposed top ranked two receptors (TLR2, USP53) and (**b**) represented the binding free energy with top ranked two independent receptors (IRF7, STAT1).

Here we have calculated the MM-PBSA binding energy for three drug agents as mentioned previously, Fig. 10a,b represents the binding energy with the top ranked two proposed (TLR2, USP53) and independent (IRF7, STAT1) receptors, respectively. On an average, USP53_Radotinib, TLR2_Torin-2 and TLR2_Rapamycin complexes produced binding energies 144.44 kJ mol^−1^, 107.97 kJ mol^−1^ and 67.64 kJ mol^−1^, respectively. On the other hand, STAT1_Radotinib, STAT1_Rapamycin and IRF7_Torin-2 complexes produced average binding energies 59.264 kJ mol^−1^, 93.333 kJ mol^−1^ and − 52.638 kJ mol^−1^, respectively.

## Discussion

The current study analyzes the high throughput RNA-Seq data to identify key genomic biomarkers (hub DEGs/proteins) highlighting their GO terms and KEGG pathways, key regulatory components (TFs and miRNAs), associated comorbidities and repurposable drugs for the treatment against SARS-CoV-2 infections by using the integrated bioinformatics approaches that were summarized in Fig. 1. Totally 109 DEGs were identified between SARS-CoV-2 infected and control samples; among them 16 upregulated and 91 down regulated genes (Table 2) were finally reported. Among them 107 DEGs encoded proteins were used to construct the PPI network (Fig. 3) which revealed ten hub-DEGs/proteins (SNRPD2, ZFP36, NEDD9, KLF6, USP53, IGF2, TLR2, PAG1, GUCY1A2 and CXCL2) which are considered as the key genomic biomarkers for SARS-CoV-2 infections.

The GO functional enrichment analysis of the proposed hub-DEGs/proteins and all the DEGs reflected the significant molecular functions that are highly linked with the COVID-19 infection and proliferation in host cell (Table 3 and Supplementary Files S1 and S2). Among the enriched MFs, the lipopolysaccharide (LPS) immune receptor activity driven by TLR2 hub-DEG is associated with the LPS-induced production of pro-inflammatory cytokines reduction, inflammation by affecting the lungs LPS due to the COVID-19 infection^56–58^. The most important and significant functions namely, cytokine regulation, produces the unnecessary “cytokine storm" that promote the adverse events like alveolar damage and fibrosis^59,60^ on COVID-19 patients. The interleukin (IL) regulatory pathways are crucial for the important pathophysiological mechanisms called systemic inflammation and cytokine release syndrome^61–63^ which were significantly associated with the hub genes (Supplementary File S1). The C–C chemokine binding functions driven by ZFP36 hub-DEG are directly involved with the T-cell induced pathogen burden controlling which is also an important receptor group protein for COVID-19^63–65^. The NAD + nucleotidase MF (steered by TLR2 hub-DEG) was found to have protective roles, and mitigate the disease severity if administered prophylactically, and its anti-hyper inflammation properties^66,67^ and the Dcp1–Dcp2 complex (steered by ZFP36 hub-DEG) play a positive role in viral infection^68^. The above enrichment analysis noticeably focuses on the association of the identified hub proteins with the diverse significant functions that are crucial for COVID-19. Moreover, the functional enrichment and pathway analysis of all the identified DEGs were recorded, and it was found that the functional pathways enriched by the key DEGs were also enriched by all the DEGs significantly (Supplementary File S1 and S2). The common functional pathways enrichment showed the biological uniformity characteristics among the all identified DEGs and the hub-DEGs.

The KEGG pathway analysis of the proposed hub-DEGs/proteins showed some enriched significant pathways. The top 10 significant KEGG pathways included the legionellosis related pathway, IL-17 signaling pathway, Rheumatoid arthritis pathway, PI3K-Akt signaling pathway, Kaposi’s sarcoma-associated herpesvirus infection and the proteoglycans in cancer pathways (Fig. 4). One of the most important pathways driven by TLR2 and CXCL2 hub-DEG namely, the legionellosis which is a typical pneumonia and exposes the cough, shortness of breath, high fever, muscle pains, and headaches^69^. These symptoms are also highly related and most common symptoms for COVID-19 positive patient^70^. During this pandemic situation, the emergency COVID-19 positive patients were permitted to treat with the Hydroxychloroquine (HCQ) although its molecular mechanisms were not completely known and later on WHO advised to avoid this for the treatment. The HCQ -is also commonly used in rheumatic disease treatment and it has been shown that the patients with rheumatoid arthritis (RA) represent lower risk of COVID-19 infection^64^. In our analysis, the hub-DEGs TLR2 and CXCL2 significantly enriched the rheumatoid arthritis pathway which indicates that these genes may have the antagonized property against the COVID-19 infection. The other significant pathways are Kaposi’s sarcoma-associated herpesvirus infection (steered by ZFP36 and CXCL2), PI3K-Akt signaling pathway and the proteoglycans in cancer pathways (steered by IGF2 and TLR2). The Kaposi’s sarcoma-associated herpesvirus infection is associated with the lung infection^71^, and the PI3K-Akt signaling pathway mainly works with the cell cycle and also with the various proteins function^72,73^. On the other hand, the proteoglycans in cancer pathways is treated as an important cancer related pathway in human^74^. Therefore, based on the molecular pathway enrichment analysis, it can be presumed that the proposed hub proteins may have significant roles in SARS-CoV-2 infection and proliferation and may be treated as prominent therapeutic target.

The TFs versus Hub-DEGs interaction network analysis revealed 6 key TFs-proteins (FOXC1, GATA2, SRF, FOXL1, YY1 and NFIC) as the transcriptional regulatory factors of hub-DEGs (Fig. 5). The basal-like breast cancer (BLBC), Alzheimer’s disease, tissue invasion are highly associated with the FOXC1 protein^75,76^. The GATA2 protein is associated with breast and kidney cancer related pathway, when the higher expression pattern of YY1 protein increases the tumour size, higher TNM stage^77–79^, the FOXL1 TF are related with proliferation, cell-cycle^80^. The SRF protein is associated with the regulation of cell survival and cell cycle progression in cardiac fibroblasts^81^. The NFIC protein has greater involvement with the tumor genesis of breast cancer, gastric cancer, and glioma^82–84^. Also the identified TFs proteins has a significant involvement in various biological functions and pathways^19–22,85^. The miR-107, miR-16-5p, miR-103a-3p, miR-27a-3p, miR-155-5p and miR-1-3p were identified as the key post transcriptional regulatory factors of hub-DEGs (Fig. 6). The miR-107 (microRNA) has direct interaction with the Coxsackie B3 virus (CVB3) replication and release^86^. The miR-16-5p represented higher expression pattern in lung cancer cell^87^ and the miR-103a-3p and miR-27a-3p has a positive correlation with the renal inflammatory dysfunction, cell proliferation and apoptosis^88,89^ while the miR-155-5p is associated with breast cancer^90^ and the miR-1-3p has the probable interaction with the SARS-CoV2^91^. The above discussion gives the evidence that the proposed regulatory TFs and the miRNAs have an enormous correlation with various biological functions and processes that are closely connected with the symptoms of SARS-CoV-2 infections and proliferation process.

The diseases versus hub-DEGs interaction network analysis showed that the predicted hub-DEGs are associated with various types of cancers and other complex diseases including the respiratory cases (Fig. 7, Supplementary Table S2). The IGF2 was connected with maximum number of diseases in the network followed by the other hub-DEGs. We observed that IGF2 gene is associated with 120 diseases including Cardiovascular Diseases, Colorectal Neoplasms, Cardiomyopathies, Liver carcinoma, Anemia; the CXCL2 was associated with 19 diseases including Rheumatoid Arthritis, Heart failure, Hypertensive disease, Inflammation, Pulmonary Fibrosis, Acute Lung Injury while the ZFP36, KLF6, GUCY1A2 and PAG1 was linked with 18 diseases including Liver Cirrhosis, Experimental Prostatic Neoplasms, Stomach Carcinoma, Inflammation, Arthritis, especially which could be a severe comorbidities of the COVID-19 patients. Among the associated diseases six diseases were connected with two hub-DEGs, notably, inflammation, mammary neoplasms, myocardial ischemia, colorectal neoplasms, somatic mutation, schizophrenia. The inflammation is considered as vital COVID-19 related comorbidity while others are also crucial. The hub-DEGs those are related with the above comorbidities also play a significant role for these diseases during the COVID-19 affection. The association of hub-DEGs with several diseases is also supported by the literature review. For example, the hub-protein SNRPD2 is significantly associated with histologic grade in Hepatocellular carcinoma (HCC), mild cognitive impairment (MCI) and Alzheimer's disease (AD)^92,93^. The hub-protein ZFP36 is associated with breast cancer and tumor-suppressive actions during hepatic tumor progression^94,95^. The hub-protein NEDD9 is significantly associated with head and neck and lung cancers^96,97^. The hub-protein KLF6 has a direct involvement in ovarian cancer cell proliferation and metastasis promotion and also works as a critical regulator of pathogenic myeloid cell activation in human^98,99^. The USP53 genes has a greater involvement in cholestatic liver disease^100,101^ and the IGF2 proteins are widely known as the diabetes associated protein and can control the insulin secretion in β-cells during fasting^102^. However, many genes/proteins related to lung disease including cancer is highly interacting with SARS-CoV-2 infections, since the patients suffer from the major complexities when the virus infects the lung. The idiopathic pulmonary fibrosis (IPF) is treated as one of the most crucial and serious risk factors of COVID-19^103^, since the COVID-19 positive patients have a greater chance for being enhanced with the IPF which creates numerous complications and leads a high risk to recover from COVID-19^104,105^. The TLR2 is highly responsive in immune-enhancing activity^106^ and the Type 2 lung inflammation is associated with the PAG1 gene expression^107^.

The multivariate survival analysis for lung cancer patients is based on low and high expressions of hub-DEGs significantly differentiated the survival curves (Fig. 7b), which indicates that the lung cancer is significantly influenced by the hub-DEGs of SARS-CoV-2 infection. Also, the patients with the lung cancers belong to the high risk of mortality from COVID-19 infection. The above discussion indicates that the proposed genomic biomarkers responsible for SARS-CoV-2 infection are also associated with various comorbidities including diabetes, lung diseases, and respiratory disease and immune systems. Therefore, covid-19 patients usually suffer from multiple complexities and reach to the severe condition that has complex comorbidities^108–110^.

To explore effective drugs for the treatments against SARS-CoV-2 infections with comorbidities, we considered the proposed human genomic biomarkers guided 10 hub-proteins (SNRPD2, ZFP36, NEDD9, KLF6, USP53, IGF2, TLR2, PAG1, GUCY1A2, and CXCL2) and 5 key TFs proteins (FOXC1, GATA2, SRF, FOXL1, and YY1) as the drug target receptor proteins and performed their docking analysis with the SARS-CoV-2 3CL protease-guided top ranked 90 FDA approved repurposable drugs. Then we selected top ranked 7 drugs (Torin-2, Rapamycin, Radotinib, Ivermectin, Thiostrepton, Tacrolimus, and Daclatasvir) as the candidate drugs for the treatment against SARS-CoV-2 infection, where the first two drugs showed strong binding affinities with all the target proteins (Fig. 8b). Among the identified candidate drugs, the Ivermectin and the Rapamycin were used to treat the COVID-19 affected patients although it has a lack of wide range of information about their activity against the SARS-CoV-2 virus^111^. Since Ivermectin has a potential antiviral activity, it has been used in the treatment of various virus infection including the SARS-CoV-2 treatment by dosing solely or with a combining other drugs^112,113^. Moreover, many studies suggested to use the Ivermectin as a potential therapeutic for COVID-19^114,115^. On the other hand, Rapamycin is widely used as inhibitor of protein synthesis and constrains the expression of pro-inflammatory cytokines such as IL-2, IL-6 and, IL-10^116^, therefore it has been also given for COVID-19 treatment^116,117^. Rapamycin can also interact with the spike protein of the SARS-CoV-2 and work in mTOR pathway inhibitors^118–121^. This result is indicating the consistency of therapeutic potentiality of the proposed hub proteins and TFs for the COVID-19 treatment. The remaining drugs were found as new potential drug candidates based on their binding affinity with the hub proteins and TFs. The Torin-2 is considered as a kinase inhibitor which worked in the PI3K-Akt/mTOR signaling pathway^117,119,122^, which supports our previous pathway analysis for the hub proteins. Radotinib (IY-5511) were being prescribed for the chronic myeloid leukaemia (CML)^123,124^ whereas Thiostrepton was used for acute kidney injury treatment^125^. The evidences show that the Tacrolimus has a positive inhibitory impact on the COVID-19 patients with comorbidities like kidney and liver transplantation^126,127^. Moreover, we validated these seven candidate drugs against the state-of-the-art alternatives already published top-ranked 11 independent and 8 protonated receptors and found their strong binding affinities (Fig. 8c), which indicates that the proposed drugs are effective against the state-of-the-arts alternatives SARS-CoV-2 infection causing independent receptor proteins also. Finally, we examined the stability of top-ranked three drugs (Torin-2, Rapamycin and Radotinib) by using 100 ns MD-based MM-PBSA simulations for two top ranked proposed (TLR2, USP53) and independent (IRF7, STAT1) receptors, and observed their stable performance according to the laws of physics^128,129^. Therefore, the proposed candidate drugs might play the vital role for the treatment against different variants of SARS-CoV-2 infections with comorbidities since our proposed target proteins are also associated with several comorbidities. The present study emphasises the further wet lab experimental validation for both the proposed target proteins and candidate drugs.

## Conclusion

The present study aims to explore genomic biomarkers (drug targets) for SARS-CoV-2 infections highlighting their functions, pathways, regulatory factors, associated comorbidities and candidate drugs. To achieve the goal, at first 109 DEGs between SARS-CoV-2 and control sample were detected from RNA-Seq profiles. The top ranked 10 hub-DEGs/proteins (TLR2, USP53, GUCY1A2, SNRPD2, NEDD9, IGF2, CXCL2, KLF6, PAG1 and ZFP36) were identified as genomic biomarkers by the PPI network analysis of DEGs with the NetworkAnalyst tool and STRING database. Gene-set enrichment analysis (GSEA) through the GO functional and KEGG pathway was then conducted to predict the associated functions and pathways of these genomic biomarkers. The gene regulatory network (GRN) analysis identified top ranked 5 TFs proteins (FOXC1, GATA2, SRF, FOXL1 and YY1) and 6 miRNAs (miR-107, miR-16-5p, miR-103a-3p, miR-27a-3p, miR-155-5p and miR-1-3p) as the transcriptional and post-transcriptional factors, respectively. The diseases versus genomic biomarkers interaction network analysis, survival analysis of lung cancer patients with genomic biomarkers and literature review showed that our proposed genomic biomarkers are associated with various types of comorbidities including diabetes, lung and heart diseases, respiratory disease and immune systems. Then we considered 10 hub-proteins (proposed genomic biomarkers) and 5 key TFs-proteins as the drug target receptor proteins to explore effective drugs by molecular docking analysis with the SARS-CoV-2 3CL protease-guided top 90 FDA approved anti-viral drugs. Then we selected top ranked 7 candidate drugs (Torin-2, Rapamycin, Radotinib, Ivermectin, Thiostrepton, Tacrolimus and Daclatasvir) with respect to our proposed 15 target proteins for the treatment against SARS-CoV-2 infection. Then we investigated the resistance of our proposed candidate drugs against the state-of-the-art alternatives of recently published top ranked 11 independent receptors for SARS-CoV-2 infections and observed that our proposed drugs are also effective against those independent receptors. Finally, we investigated the stability performance of top three drugs (Torin-2, Rapamycin and Radotinib) by using 100 ns MD-based MM-PBSA simulations for two top ranked proposed receptors (TLR2, USP53) and top two independent receptors (IRF7, STAT1), and observed their stable performance. In the context of already published host transcriptome-guided candidate drugs for covid-19, so far, no researchers yet investigated the resistance of their suggested drugs against the state-of-the-art alternatives independent receptors proposed by others computationally. In this study, we considered this issue. Thus, we may sate that this study is partially unique. As covid-19 is a new coronavirus disease, there has been little research on exploring globally effective drugs. In this regard, this research on coronavirus disease might open up new possibilities to explore globally more effective drugs computationally.

## Materials and methods

### Data sources and descriptions

We used both original data and metadata associated with SARS-CoV-2 infections to reach the goal of this study as described below.

### Collection of RNA-Seq profiles as original data (case/control)

We collected the original host RNA-Seq count dataset to explore genomic biomarkers and drug target key receptor proteins associated with SARS-CoV-2 infection. This dataset was downloaded from the NCBI Gene Expression Omnibus (GEO) data repository with the accession number GSE150316. This dataset consisted of 88 samples generated from different organs including lung, heart, jejunum, liver, kidney, bowel, fat, skin, bone marrow and placenta, where 5 samples were COVID-19 negative (control). The count of each sample was generated on 59,091 transcripts. This dataset was first analyzed by Desai et al.^130^ to investigate the temporal and spatial heterogeneity of host genome response to SARS-CoV-2 pulmonary infection. In our case, we considered only lung tissue samples to avoid the spatial heterogeneity problem from the dataset. Our analyzed original dataset consisted of 35 lung tissue samples infected with SARS-CoV-2 (case) and 5 control samples.

### Collection of drug agents and receptor proteins as metadata

We collected SARS-CoV-2 3CL protease-guided top listed 90 drugs out of 3410 FDA approved antiviral drugs published by Beck et al.^50^ as the meta drug agents to explore few top ranked host transcriptome-guided drugs against SARS-CoV-2 infections by molecular docking with our proposed receptor proteins. The 3D structures of 90 FDA-approved drugs (Supplementary Table S1) were downloaded from PubChem database^54^. To validate our proposed host transcriptome-guided repurposed drugs by molecular docking with the top listed receptor proteins that were published in different reputed journals, we reviewed 22 different articles infections^28–49^ associated with SARS-CoV-2 infections and selected 11 top listed receptor proteins out of 193. Then their docking performance with our proposed drugs were investigated whether the drugs are keeping consistency with high binding affinities as in our proposed drug target hub-DEGs.

### Integrated bioinformatics and system biology analyses

The integrated bioinformatics and system biology approaches were utilized in this study as described below:

### Identification of differentially expressed genes (DEGs)

Identification of differentially expressed genes (DEG) is one the most important tasks in this study. There are several methods for identification of DEGs from RNA-Seq profiles. Most of them are sensitive to outlying observations. There are a few robust approaches for identification of DEGs from RNA-Seq profiles. However, non-robust approaches are slightly better than robust approaches in absence of outliers, while the robust approaches are much better than the classical approaches in presence of outliers. Therefore, we considered edgeR^131^ as a popular non-robust and DESeq2^132^ as a popular robust approaches to take their advantages in our analyses, since few RNA-Seq counts are often contaminated by outliers due to several steps involves in the data generating process. However, normalization is a compulsory step for RNA-Seq data analysis. It removes systematic technical bias from the data and makes the samples comparable. The edgeR approach utilizes TMM (trimmed mean of M values) normalization, whereas the DESeq2 utilizes the median-of-ratios method. The edgeR method was formulated based on generalized linear model (GLM) of the negative binomial family. It assumes negative binomial (NB) distribution for the read counts and uses the empirical Bayes for squeezing the tag-wise dispersions toward common dispersion, whereas DESeq2 also considered GLM of the NB family and assumes NB distribution for the read counts and uses the empirical Bayes to shrink gene-wise dispersion estimates towards fitted values. The edgeR approach utilizes the likelihood ratio test (LRT) statistic to calculate the *p*-values and test the null hypothesis of no differential read counts between case and control groups, whereas the DESeq2 utilizes the Wald test statistic to calculate the *p*-values and test the null hypothesis of no differential read counts between case and control groups. The *p*-values of both the edgeR and the DESeq2 approaches are then adjusted for multiple testing using the procedure of Benjamini and Hochberg^133^.

In this paper, we considered the *g*th gene (*g* = 1,2, …, 59,091) as a differentially expressed gene (DEG) between case and control groups if its adjusted *p*_*g*_*-*value < 0.05 along with |log_2_(aFC_g_)|> 1 by controlling the false discovery rate (FDR) at 5%, otherwise, it was considered as equally expressed gene (EEG). The *g*th gene was considered as upregulated or downregulated DEG if its adjusted *p*_*g*_*-*value < 0.05 along with log_2_(aFC_g_) > 1 or log_2_(aFC_g_) < − 1, respectively. Here aFC is the abbreviation of average fold change which is defined as ${\text{aFC}}_{g}={\overline{x} }_{g}/{\overline{y} }_{g}$ (the fold change of ${\overline{x} }_{g}$ with respect to ${\overline{y} }_{g}$), where ${\overline{x} }_{g}$ and ${\overline{y} }_{g}$ are the averages of normalized counts of case and control groups with respect to *g*th gene, respectively. For example, a change from ${\overline{y} }_{g}=3$ to ${\overline{x} }_{g}=9$ produces aFA_g_ = 3 which is referred to as a "threefold upregulated in average". Similarly, a change from ${\overline{y} }_{g}=9$ to ${\overline{x} }_{g}=3$ produces aFA_g_ = 1/3 which is referred to as a "threefold downregulated in average". Let *A*_*UR*_ and *A*_*DR*_ were the upregulated and downregulated DEGs-sets respectively, detected by edgeR. Again, let *B*_*UR*_ and *B*_*DR*_ were the upregulated and down-regulated DEGs-sets respectively, detected by DESeq2. Then we defined upregulated gene-set as $({A}_{UR}\cup {B}_{UR})$ and down-regulated as $({A}_{DR}\cup {B}_{DR})$ by combining the results DEGs results of edgR and DESeq2. Let *C* was the set of contradictory upregulated and downregulated DEGs estimated by both edgeR and DESeq2. For example, if a DEG $g\in {A}_{UR}$ but $g\notin {B}_{UR}$ or $g\in {A}_{DR}$ but $g\notin {B}_{DR}$, then the DEG ‘*g*’ was considered as a contradictory DEG. We removed this type of contradictory DEGs from further analysis. Then the final DEGs-set for further analysis was defined as

$$DEG=\left[\left({A}_{UR}\cup {B}_{UR}\right)\cup \left({A}_{DR}\cup {B}_{DR}\right)\right]-C=[\left({A}_{UR}\cup {B}_{UR}\right)\cup \left({A}_{DR}\cup {B}_{DR}\right)]\cap {C}^{/},$$

where, upregulated DEGs-set was defined as

$${DEG}_{UR}=\left({A}_{UR}\cup {B}_{UR}\right)-C=\left({A}_{UR}\cup {B}_{UR}\right)\cap {C}^{/}$$

and the downregulated DEGs-set was defined as

$${DEG}_{DR}=\left({A}_{DR}\cup {B}_{DR}\right)-C=\left({A}_{DR}\cup {B}_{DR}\right)\cap {C}^{/}.$$

### Functional and pathway enrichment analysis of Hub-DEGs

Gene ontology (GO) functional and Kyoto encyclopedia of genes and genomes (KEGG) pathway enrichment/annotation/over-representation analysis^134^ is a widely used approach to determine the significantly annotated/enriched/over-represented functions/classes/terms (biological processes (BP), molecular functions (MF) and cellular components (CC)) and pathways by the identified DEGs/Hub-DEGs. The BP is a change or complex of changes during the granularity period of the cell that is mediated by one or more gene products for different biological objectives. The MFs are the biochemical activities of gene products. The CC is a place in a cell in which a gene product is active. KEGG pathway is a collection of experimentally validated pathway maps^135–137^ representing our knowledge of the molecular interaction, reaction and relation networks for metabolism, cellular processes, genetic information processing, organismal systems, environmental information processing, human diseases and drug development. Let *S*_*i*_ is the annotated gene-set corresponding to *i*th type of biological functions or pathways given in the database and *M*_*i*_ is the number of genes in *S*_*i*_ (*i* = 1, 2,…,*r*); *N* is the total number of annotated genes those construct the entire combine set $S=\bigcup_{i=1}^{r}{S}_{\text{i}}={S}_{i}\bigcup {S}_{i}^{c}$ such *that*$N\le \sum_{i=1}^{r}{M}_{i} ;$ where ${S}_{i}^{c}$ is the complement set of *S*_*i*_. Again, let *n* is the total number of DEGs of interest and *k*_*i*_ is the number of DEGs belonging to the annotated gene-set *S*_*i*_. This problem is summarized by the following contingency table (Table 1):

**Table 1:** Contingency table.

Gene-set (annotated)	DEGs	Not DEGs	Marginal total
*S*_*i*_ (*i*th GO term/KEGG-pathway)	*k*_*i*_	*M*_*i*_ − *k*_*i*_	*M*_*i*_
${S}_{{\varvec{i}}}^{c}$(Complement of *S*_*i*_)	*n* − *k*_*i*_	*N* − *M*_*i*_ − *n* + *k*_*i*_	*N* − *M*_*i*_
Marginal total	*n*	*N* − *n*	*N* (Grand total)

The probability of observing exactly *k*_*i*_ DEGs in *S*_*i*_ (*i*th functional/pathway annotated gene-set) out of *n* DEGs can be modeled by the hypergeometric distribution. Hence, the probability of observing *k*_*i*_ or more genes in *S*_*i*_ out of *n* DEGs can be calculated by the cumulative probability as follows

$${p}_{i}=1-{\sum }_{j=0}^{{k}_{i}}\frac{\left(\genfrac{}{}{0pt}{}{Mi}{j}\right)\left(\genfrac{}{}{0pt}{}{N-Mi}{n-j}\right)}{\left(\genfrac{}{}{0pt}{}{N}{n}\right)}, i=\text{1,2},\dots ,r.$$

The subset of DEGs belonging to *S*_*i*_ is said to be significantly enriched if its adjusted *p*-value (*p*_*i*_) is less than 0.05 by controlling the FDR at 5%. The g:GOSt core, a free web tool for functional analyses (https://biit.cs.ut.ee/gprofiler/gost), embedded with the g:Profiler web server^138^ utilizes the cumulative hypergeometric approach to calculate these *p*-values (*p*_*i’*_*s*) and their adjusted *p*-values for multiple testing using the procedure of Benjamini and Hochberg^133^. The g:Profiler is a regularly updated database for performing GO functional and KEGG pathway enrichment analysis. Therefore, in this paper, we performed functional and pathway enrichment analysis by using the g:GOSt tool entrenched in the g:Profiler web server to disclose the statistically significant GO terms of biological processes, molecular functions and cellular components and KEGG pathways for DEGs associated with the SARS-CoV-2 infections.

### Protein–protein interaction (PPI) network analysis of DEGs

Protein–protein interactions (PPIs) are the physical magnetism of two or more protein molecules that occur due to biochemical reactions steered by hydrogen bonding, electrostatic forces and the hydrophobic effect. Generally, a protein cannot work without interaction with one or more other proteins. The PPI network contributes to the formation of larger protein complexes for performing a specific task^139^. It carries out many molecular functions and biological processes including protein function, cell-to-cell interactions, metabolic and developmental control, disease incidence, and therapy design. A PPI network is represented as an undirected graph, where nodes and edges indicate proteins and their interactions, respectively. A node having the largest number of significant interactions/connections/edges with other nodes is considered as the top ranked hub-protein. Therefore, the PPI network analyses of DEGs are now widely using to explore hub-DEGs/proteins. In this study, the PPI network of DEGs was constructed by using the NetworkAnalyst^140^ with the STRING database^141^ plagued in Cytoscape. A topological exploration based on dual-metric measurements degree (> 10) and betweenness were utilized to determine the highly representative DEGs/proteins those are also known as hub-DEGs/proteins.

### Regulatory network analysis of hub-DEGs

A gene regulatory network (GRN) shows molecular regulators that interact with each other to control the gene expression levels of mRNA and proteins. Transcription factors (TFs) and microRNAs (miRNAs) are considered as the most important molecular regulators of genes. A transcription factor (TF) is a protein that binds to a specific DNA region (promoter/enhancer) and regulates gene expression by promoting or suppressing transcription. TFs are considered as the main players in GRN. A miRNA is a small single-stranded non-coding RNA molecule (containing about 22 nucleotides) that works in RNA silencing and post-transcriptional regulation of gene expression. There are up to 1600 TFs and 1900 miRNAs in the human genome. A TFs and hub-DEGs/proteins interaction network is considered as an undirected graph, where nodes indicate TFs or hub-DEGs and edges represents interactions between TFs and hub-DEGs, respectively. A TF-node having the largest number of significant interactions/connections/edges with hub-DEGs nodes is considered as the top ranked hub-TF regulator of hub-DEGs. To explore hub-TFs of hub-DEGs, we contracted the interaction network between TFs and hub-DEGs by using the NetworkAnalyst tool^140^ based on JASPAR database^142^. Similarly, miRNA and hub-DEGs interaction network was constructed through the NetworkAnalyst based on TarBase *V8.0*^143^ database to identify the key regulatory miRNAs for hub-DEGs. These key regulatory biomolecules were selected based on the highest topological matrices (degree of connectivity and betweenness) applied on the interaction network.

### Association of hub-DEGs with comorbidities

To investigate the association of hub-DEGs with other diseases, we performed diseases versus hub-DEGs interaction network analysis by using the NetworkAnalyst tool^140^ based on DisGeNET^144^ database. We also performed survival analysis based on the expression of hub-DEGs with lung cancer patients by using Kaplan–Meier (KM) plotter^145^ to investigate the association of hub-DEGs with lung cancer, since SARS-CoV-2 samples were collected from lung tissue and also it affects the lung mostly. The KM plotter utilizes the log rank statistic to test the significance of association.

### Drug repurposing by molecular docking study

To propose in-silico validated efficient FDA approved repurposed drugs for the treatment of SARS-CoV-2 infections, we employed molecular docking between the target receptor proteins and drug agents. We considered our proposed hub-DEGs based hub-proteins and associated TFs proteins as the drug target receptor proteins and SARS-CoV-2 transcriptome-guided top listed 90 drugs out of 3410 FDA approved antiviral drugs published by Beck et.al. 2020^50^ as drug agents or ligands for docking analysis. The molecular docking study requires 3-Dimensional (3D) structures of both receptor proteins and candidate drugs. We downloaded the 3D structure of all targeted receptor proteins from Protein Data Bank (PDB)^51^ and SWISS-MODEL^52^, a homology modeling based database. The 3D structures of our selected 90 drug agents (Supplementary Table S1) were downloaded from PubChem database^54^. The 3D structure of the target proteins was visualized using Discovery Studio Visualizer 2019^146^ and the water molecules, co-crystal ligands which were bound to the protein were removed. Further, the protein was prepared using USCF Chimera and Autodock vina^147^ in PyRx open source software by adding charges and minimizing the energy of the protein and subsequently converting it to pdbqt format^147–149^. The exhaustiveness parameter was set to 8. The Protein–Ligand Interaction Profiler (PLIP) web service^150^ and PyMol^151^ were used to analyze the docked complexes for surface complexes, types and distances of non-covalent bonds. Let *A*_*ij*_ denotes the binding affinity between *i*th target protein (*i* = 1, 2, … , *m*) and *j*th drug agent (*j* = 1, 2, … , *n*). Then target proteins are ordered according to the descending order of row sums ${\sum }_{j=1}^{n}{A}_{ij}$, *j* = 1,2, … , *m*, and drug agents are ordered according to the descending order of column sums ${\sum }_{i=1}^{m}{A}_{ij}$, *j* = 1,2, … , *n*, to select the top ranking few drug agents as the candidate drugs. The average binding affinity score less than or equal to − 8.0 was considered as the better drug selection criterion against the receptor proteins. Then we validated the proposed repurposed drugs by molecular docking with the top listed receptor proteins associated with SARS-CoV-2 infections that were obtained by the literature review. To select the top listed receptor proteins associated with SARS-CoV-2 infections, we reviewed 22 recently published articles^28–49^ and selected the top listed 11 receptor proteins.

### Molecular dynamic (MD) simulations

MD simulations were carried out by using YASARA Dynamics software^152^, and the AMBER14 force field^153^ to study the dynamic behavior of the top-ranked protein–ligand complexes. A total of six different systems were used to run MD simulation. The systems included top three hits, TLR2_Torin-2, TLR2_Rapamycin, USP53_Radotinib complexes corresponding to our proposed receptors and another three hits, IRF7_Torin-2, STAT1_Rapamycin, STAT1_Radotinib complexes from top listed independent receptors. Before simulation, the target-drug complex's hydrogen bonding network was optimized and solvated by a TIP3P^154^ water model in a simulation cell. Periodic boundary conditions were maintained with a solvent density of 0.997 g L^ −1^. Titratable amino acids in the protein complex were subjected to pKa calculation during solvation. The initial energy minimization process of each simulation system, consisting of 41,645 ± 10, 41,645 ± 10, 95,924, 105,924 ± 10, 105,924 ± 10 and 85,924 atoms for TLR2_Torin-2, TLR2_Rapamycin, USP53_Radotinib, STAT1_Rapamycin, STAT1_Radotinib and IRF7_Torin-2 complexes was performed by a simulated annealing method respectively, using the steepest gradient approach (5000 cycles). Each simulation was run with a multiple time step algorithm^155^, using a time-step interval of 2.50 fs under physiological conditions (298 K, pH 7.4, 0.9% NaCl)^156^. All bond lengths were constrained using the linear constraint solver (LINCS)^157^ algorithm, and SETTLE^158^ was used for water molecules. Long-range electrostatic interactions were described by the PME^159^ methods, and, finally, 100 ns MD simulation was accomplished at Berendsen thermostat^160^ and constant pressure. The trajectories were recorded every 250 ps for further analysis, and subsequent analysis was implemented by default script of YASARA^161^ macro and SciDAVis free software available at http://scidavis.sourceforge.net/. All snapshots were then subjected to YASARA software's MM-PBSA (MM-Poisson–Boltzmann surface area) binding free energy calculation using the formula below^162^,

$$\text{Binding free Energy}= {\text{E}}_{\text{potReceptor}}+{\text{E}}_{\text{solvReceptor}}+ {\text{E}}_{\text{potLigand}}+ {\text{E}}_{\text{solvLigand}}- {\text{E}}_{\text{potComplex}}- {\text{E}}_{\text{solvComplex}}.$$

Here, MM-PBSA binding energy was calculated using YASARA built-in macros using AMBER 14 as a force field, with larger positive energies indicating better binding^163^. The PBSA is one of the most appealing solvation systems used in computer-aided drug design techniques to determine binding energy of protein–drug complexes^164,165^.

## Supplementary Information

Supplementary Information 1.

Supplementary Information 2.

Supplementary Information 3.

Supplementary Information 4.
