# Supplementary material for: Computational identification of host genomic biomarkers highlighting their functions, pathways and regulators that influence SARS-CoV-2 infections and drug repurposing
Source: Sci Rep. 2022 Mar 11;12:4279. doi: 10.1038/s41598-022-08073-8 (PMC8915158; doi:10.1038/s41598-022-08073-8)
Supplement: Supplementary file 3 — Supplementary Information 3. [file 41598_2022_8073_MOESM3_ESM.pdf]

Supplementary File 3 (File-S3)

This file contains Table S1, Table S2, Table S3 and Table S4.

Table S1: FDA approved top listed 90 anti-viral drugs for SARS-CoV-2 infections proposed by Beck et.al. 2020 [47]

5-nonyloxytryptamine	eprosartan	Rapamycin (Sirolimus)
Abacavir sulfate	Etomidate	Remdesivir
Abacavir	everolimus	ribavirin
Acetylcholine Chloride	Famciclovir	Rifabutin
Acyclovir	foxy-5	Rilpivirine
Adefovir Dipivoxil	Ganciclovir	Ritonavir
Amprenavir (agenerase)	indinavir	Rupatadine Fumarate
Apixaban	ivermectin	Saquinavir mesylate
Asunaprevir (BMS-650032)	Leuprolide Acetate	saquinavir
Atazanavir sulfate (BMS-232632-05)	lisuride	saracatinib
Atazanavir	lopinavir	scopolamine
Atropine	Methscopolamine	Sildenafil Citrate
avermectin	mupirocin	sirolimus
Batimastat	naltrindole	somatostatin
Boceprevir	Nelfinavir Mesylate	Tacrolimus (FK506)
bosutinib	nelfinavir	Telaprevir (VX-950)
Cidofovir	nevirapine	temsirolimus
Cyclosporine	Octreotide acetate	Tenofovir Disoproxil Fumarate
dacinostat	oligomycin-a	tenofovir
Daclatasvir (BMS-790052)	Oseltamivir acid	thiostrepton
danoprevir	Oseltamivir phosphate	Tigecycline
Daptomycin	Oseltamivir	Tiotropium Bromide
Darunavir	Otilonium Bromide	torin-2
demecarium	Penciclovir	trichostatin-a
Difloxacin HCl	Peramivir Trihydrate	Valaciclovir HCl
dinoprostone	Peramivir	valaciclovir
efavirenz	Pimecrolimus	Valganciclovir HCl
elvitegravir	prostaglandin	Zanamivir
Entecavir Hydrate	Radotinib(IY-5511)	zolmitriptan
entecavir	raltegravir	PHA-665752

Table S2: The list of diseases that are significantly associated hub-DEGs detected by the hub-DEGs versus Diseases interaction network analysis.

Hub-DEGs	Diseases	Hub-DEGs	Diseases
CXCL2	Rheumatoid Arthritis	IGF2	Bulging forehead
CXCL2	Bone Resorption	IGF2	Increased incidence of hepatocellular carcinoma
CXCL2	Cholestasis	IGF2	Liver carcinoma
CXCL2	Esophageal Neoplasms	IGF2	Feeding difficulties in infancy
CXCL2	Heart failure	IGF2	Fetal overgrowth
CXCL2	Hypertensive disease	IGF2	Isolated somatotropin deficiency
CXCL2	Inflammation	IGF2	No development of motor milestones
CXCL2	Chronic Obstructive Airway Disease	IGF2	Mental and motor retardation
CXCL2	Degenerative polyarthritis	IGF2	Large bregma sutures
CXCL2	Pulmonary Fibrosis	IGF2	Wide bregma sutures
CXCL2	Reperfusion Injury	IGF2	Thickened facial skin with coarse facial features
CXCL2	Dermatologic disorders	IGF2	Noncancerous mole
CXCL2	Myocardial Ischemia	IGF2	Curvature of little finger
CXCL2	Acute Lung Injury	IGF2	Prominent back of the head
CXCL2	Arsenic Poisoning	IGF2	Somatic mutation
CXCL2	Mammary Neoplasms	IGF2	Colorectal Neoplasms
CXCL2	Dermatitis, Allergic Contact	IGF2	Anemia
CXCL2	Infarction, Middle Cerebral Artery	IGF2	Atherosclerosis
CXCL2	Shock, Hemorrhagic	IGF2	Autistic Disorder
GUCY1A2	Mammary Neoplasms	IGF2	Beckwith-Wiedemann Syndrome
GUCY1A2	Colorectal Neoplasms	IGF2	Cardiovascular Diseases
IGF2	Congenital hemihypertrophy	IGF2	Cognition Disorders
IGF2	Short stature	IGF2	Colonic Neoplasms
IGF2	Large fontanelle	IGF2	Craniopharyngioma
IGF2	Delayed bone age	IGF2	Cryptorchidism
IGF2	Blue sclera	IGF2	Exophthalmos
IGF2	Enlarged kidney	IGF2	Fetal Growth Retardation
IGF2	Advanced bone age	IGF2	Foot Deformities
IGF2	Global developmental delay	IGF2	Growth Disorders
IGF2	prenatal alcohol exposure	IGF2	Cardiomegaly

IGF2	Acquired scoliosis	IGF2	Hepatomegaly
IGF2	Lewy Body Disease	IGF2	Diaphragmatic Hernia
IGF2	Congenital omphalocele	IGF2	Polyhydramnios
IGF2	Cardiomyopathies	IGF2	Hypoglycemia
IGF2	Weight decreased	IGF2	Liver neoplasms
IGF2	Facial asymmetry	IGF2	Low Birth Weights
IGF2	Intrauterine retardation	IGF2	Macroglossia
IGF2	Penile hypospadias	IGF2	Memory Disorders
IGF2	Short middle phalanx of the 5th finger	IGF2	Micrognathism
IGF2	Triangular face	IGF2	Mild Mental Retardation
IGF2	Short distal phalanx	IGF2	Muscle hypotonia
IGF2	Prominent forehead	IGF2	Nephroblastoma
IGF2	Prominent eyes	IGF2	Nerve Degeneration
IGF2	Ureteral anomalies	IGF2	Melanocytic nevus
IGF2	Weight less than 3rd percentile	IGF2	Obesity
IGF2	Coarse facial features	IGF2	Parkinson Disease
IGF2	X- linked recessive	IGF2	Placenta Disorders
IGF2	Protruding eyes	IGF2	Precancerous Conditions
IGF2	Relative macrocephaly	IGF2	Rhabdomyosarcoma
IGF2	Overgrowth	IGF2	Schizophrenia
IGF2	Bilateral fifth finger clinodactyly	IGF2	Seminoma
IGF2	Adrenocortical cytomegaly	IGF2	Curvature of spine
IGF2	Overgrowth of external genitalia	IGF2	Syndactyly
IGF2	Generalized overgrowth	IGF2	Tooth Abnormalities
IGF2	Pancreatic hyperplasia	IGF2	Underweight
IGF2	Isolated cases	IGF2	Neonatal hypoglycemia
IGF2	Prominent occiput	IGF2	Russell-Silver syndrome
IGF2	Motor delay	IGF2	Hepatoblastoma
IGF2	Hypoplastic mandible condyle	IGF2	Gonadoblastoma
IGF2	Asymmetric chest	IGF2	Adrenocortical carcinoma
IGF2	Prominent globes	IGF2	Cafe-au-Lait Spots
IGF2	Increased hepatocellular carcinoma risk	IGF2	Frontal bossing
IGF2	Cognitive delay	KLF6	Liver Cirrhosis, Experimental
IGF2	Downturned corners of mouth	KLF6	Prostatic Neoplasms
IGF2	Craniofacial disproportion	KLF6	Stomach Neoplasms
IGF2	Birthmark	KLF6	Malignant neoplasm of prostate
IGF2	Congenital ear anomaly	KLF6	Somatic mutation

	NOS		
IGF2	Dental abnormalities	KLF6	Stomach Carcinoma
IGF2	Fasting Hypoglycemia	PAG1	Schizophrenia
IGF2	Late fontanel closure	PAG1	Precursor Cell Lymphoblastic Leukemia Lymphoma
IGF2	Bone Diseases, Developmental	ZFP36	Inflammation
IGF2	Infant, Small for Gestational Age	ZFP36	Myocardial Ischemia
IGF2	Large, late-closing fontanelle	ZFP36	Alopecia
IGF2	Diastasis recti	ZFP36	Arthritis
IGF2	Small for gestational age	ZFP36	Cachexia
IGF2	Congenital posterior urethral valves	ZFP36	Dermatitis
IGF2	Low set ears	ZFP36	Hyperplasia
IGF2	Mandibular hypoplasia	ZFP36	Neoplasm Invasiveness
IGF2	Cardiovascular Abnormalities		

Table S3: Summary of candidate drugs against hub proteins for COVID-19 using molecular docking simulation strategy. Five candidate drugs were selected by investigating the binding affinity score including, including Torin-2, Ivermectin, Radotinibiy (5511), Rapamycin (Sirolimus), Thiostrepton. The 3D structure of hub protein with candidate drugs are shown in 2nd column. The drug interactions with hub protein is given 3rd column and neighbor residues (within 4 Å of the drug) are shown. Interacting amino acids and their types are shown in the last column.

The name of Complex	The 3d view of complex	Ligand interactions 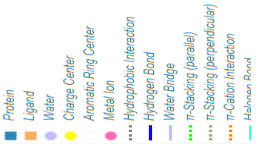	Interacting amino acids			
			Hydrogen bond	Hydrophobic Interactions	Halogen/ Salt Bridge	π-Stacking
CXCL2_TORIN-2	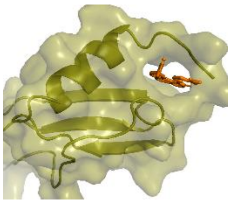	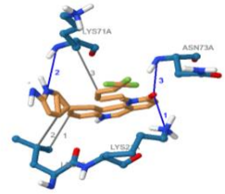	Lys20, Lys71, Asn73	Leu20, Lys 71	-	-

IGF2_TORIN-2	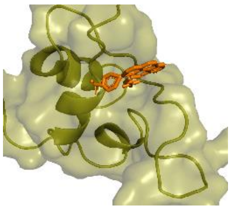	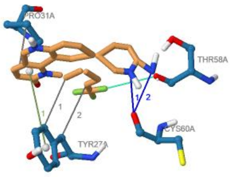	Cys60	Tyr27, Pro31	Thr58	Tyr27
KLF6_TORIN-2	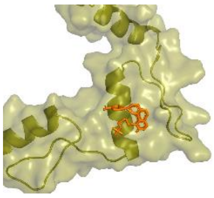	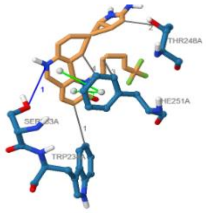	Ser233	Trp234,Thr248, Phe251	-	Phe251
NEDD9_RADOTINIBIY_5511	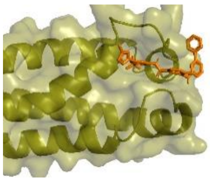	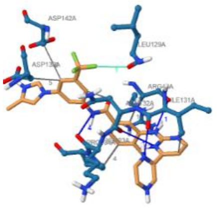	Arg49, Ile131, Asn132, Lys133	Arg49, Ile131, Asn132, Pro134, Asp139, Asp142	Leu129	-
PAG1_TORIN-2	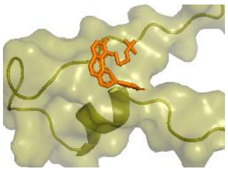	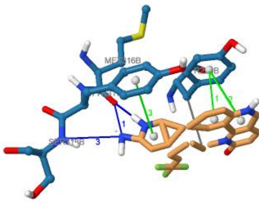	Met316, Ser318	Tyr299	-	Tyr29, Tyr317
SNRPD2_TORIN-2	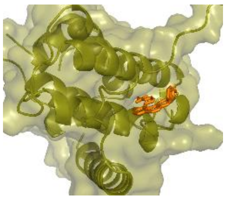	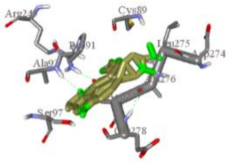	Ala92, Arg245, Ala276, Asp277, Glu278	Cys89, Pro91, Ala92, Asp274	-	-
USP53_THIOSTREPTON	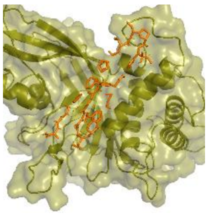	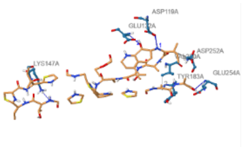	Asp119, Glu122, Lys147, Glu254	Tyr183, Val250, Asp252	-	-
ZFP36_RAPAMYCIN_SIROLIMUS	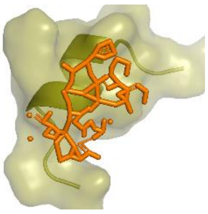	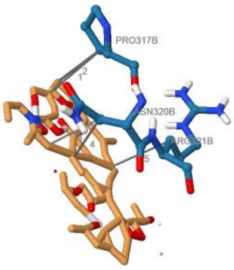	-	Pro317, Asn320, Arg321	-	-

Table S4: Summary of candidate drugs against key transcription factors (TFs) for COVID-19 using molecular docking simulation strategy. Three candidate drugs were selected by investigating the binding affinity score including, including Torin-2, Rapamycin (Sirolimus), Tacrolimus (FK506). The 3D structure of hub protein with candidate drugs are shown in 2nd column. The drug interactions with hub protein is given 3rd column and neighbor residues (within 4 Å of the drug) are shown. Interacting amino acids and their types are shown in the last column.

The name of Complex	The 3d view of complex	Ligand interactions 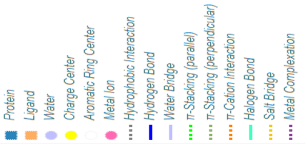	Interacting amino acids: Hydrogen bond	Hydrophobic Interactions	Halogen/ Salt Bridge	π-Stacking
FOXC1_RAPAMYCIN (SIROLIMUS)	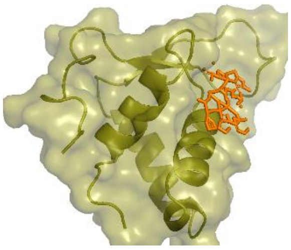	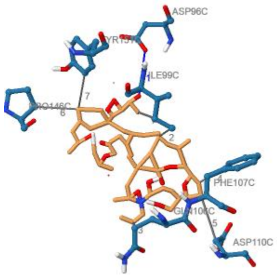	Asp96	Ile99, Gln106, Phe107, Asp110, Pro146, Tyr151	-	-
FOXL1_TORIN-2	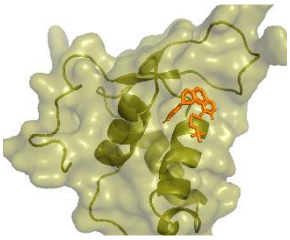	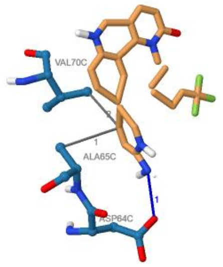	Asp64	Ala65, Val70	-	-
GATA2_TORIN-2	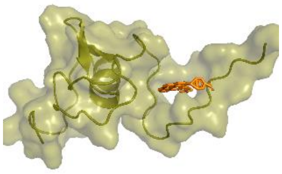	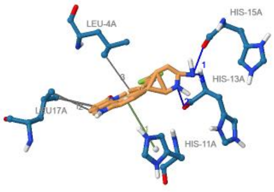	His13, His15	Leu4, Leu17	-	His

SRF_TACROLIMUS (FK506)	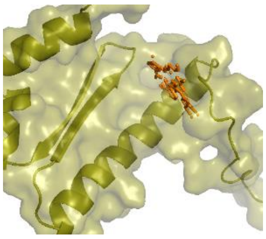	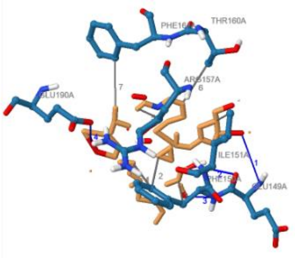	Glu149, Phe150, Glu190	Phe150, Ile151, Arg157, Thr160, Phe161	-	-
YY1_TORIN-2	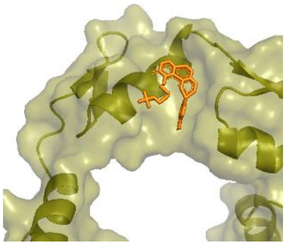	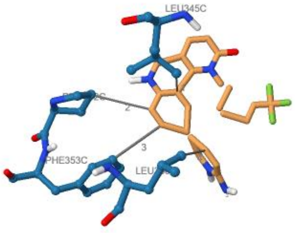	-	Leu345, Pro352, Phe353, Leu366	-	-
